# Intra-Session recurrence in intradialytic hypotension prediction: evaluation implications and recurrence-aware modeling

**DOI:** 10.3389/fdgth.2026.1804788

**Published:** 2026-07-07

**Authors:** Siun Kim, Jiwon Ryu, Sejoong Kim, Su Hwan Kim, Myeongju Kim, Hyung-Jin Yoon

**Affiliations:** 1 Biomedical Research Institute, Seoul National University Hospital, Seoul, Republic of Korea; 2 Seoul National University Bundang Hospital, Seongnam-si, Republic of Korea; 3 Department of Internal Medicine, Seoul National University College of Medicine, Seoul, Republic of Korea; 4 Department of Information Statistics, Gyeongsang National University, Jinju, Republic of Korea; 5 Center for Artificial Intelligence in Healthcare, Seoul National University Bundang Hospital, Seongnam, Republic of Korea; 6 Department of Human Systems Medicine, Seoul National University College of Medicine, Seoul, Republic of Korea; 7 Medical Bigdata Research Center, Seoul National University College of Medicine, Seoul, Republic of Korea

**Keywords:** adversarial training, deep learning, hemodialysis monitoring, IDH recurrence, integrated gradient method, intradialytic hypotension, real-time risk prediction

## Abstract

**Background:**

Real-time prediction of intradialytic hypotension (IDH) using deep learning has shown high accuracy; however, existing models typically treat all IDH events equally, without distinguishing between initial and recurrent occurrences within a dialysis session. This conventional approach neglects the distinct underlying physiological mechanisms and clinical intervention requirements of each occurrence type. This study aimed to systematically examine IDH recurrence patterns to evaluate their impact on model performance and to identify methodological improvements.

**Methods:**

We retrospectively analyzed 12,767 hemodialysis sessions from 66 patients. Recurrent IDH was defined as an event occurring ≥30 min after the initial IDH. Deep learning models, including ConvMixer, temporal convolutional network, and long short-term memory with attention, were compared with a rule-based naïve baseline that predicted IDH solely from prior occurrence. Modeling strategies explicitly incorporating recurrence information were implemented. Model robustness across systolic blood pressure subgroups was evaluated and enhanced using adversarial training.

**Results:**

The probability of IDH increased markedly from 0.7–10.4% before initial events to 11.7–65.7% thereafter. Conventional evaluation that aggregated all IDH events overestimated performance, with particularly large gaps in F1 score and AUPRC between initial and recurrent IDH predictions. The naïve baseline achieved an area under the receiver operating characteristic (AUROC) curve of 0.798 without training, highlighting the strong influence of recurrence patterns on predictive performance. Incorporating recurrence-aware features and loss weighting improved AUROC by up to 8.0 percentage points across architectures. Adversarial training further reduced subgroup disparities while preserving overall model performance.

**Conclusions:**

Incorporating recurrence patterns into IDH prediction models demonstrated improvements in accuracy, robustness, and comparability across studies. We recommend standardized evaluation protocols that explicitly account for recurrence to enhance clinical applicability and reliability.

## 1 Introduction

Intradialytic hypotension (IDH) is a common complication during hemodialysis, often causing symptoms such as dizziness, fatigue, and arrhythmia (1). Frequent IDH occurrence can significantly impair quality of life, increase the risk of ischemic organ injury, and elevate mortality rates in patients undergoing dialysis (2–6). The reported prevalence of IDH varies widely across studies, ranging from 4.0% to 31.0% (7, 8), with a recent meta-analysis reporting a pooled estimate of 31% (95% CI, 18%–44%) among hospital-based renal replacement therapy cohorts (9), largely owing to the lack of a standardized definition. Current definitions of IDH focus on diverse aspects, such as nadir blood pressure (BP) levels (10), decrease in BP from baseline (6, 11, 12), symptoms, or necessary clinical interventions (13, 14). This lack of consensus hinders the comparison of research findings across studies and impedes the development of standardized prevention and intervention protocols (9).

Deep learning techniques have shown promise in predicting IDH using patient data collected before and during dialysis (15–23). These approaches include pre-session prediction (15, 16) using pre-dialytic patient information and intra-session or real-time prediction (17–20). Real-time IDH prediction models aim to forecast IDH at predefined intervals (e.g., 1 h or 10 min) before its occurrence, providing timely alerts for intervention (20, 21). These models have achieved high performance, with area under the receiver operating characteristic curve (AUROC) scores exceeding 0.85 (17, 20) and reaching up to 0.95 (19).

However, previous studies have developed and evaluated real-time IDH prediction models using all IDH occurrences during dialysis without distinguishing between initial and recurrent IDH events. This conventional approach fails to adequately consider the recurrence patterns of IDH events within dialysis sessions, potentially affecting the interpretation of model performance and clinical utility.

To address this issue, our study aimed to (i) investigate the recurrence patterns of IDH events within single dialysis sessions across different IDH definitions, (ii) assess how these recurrence patterns influence the performance of real-time IDH prediction models, (iii) explore various modeling strategies to incorporate IDH occurrence information and enhance predictive performance; and (iv) provide recommendations for the development, evaluation, and performance reporting of real-time IDH prediction models.

## 2 Materials and methods

### 2.1 Data source and study population

We conducted a retrospective analysis of data from a hemodialysis monitoring program at Seoul National University Bundang Hospital from July 2022 to January 2024. This study included 12,767 hemodialysis sessions from 66 outpatients aged 18 years and above, all with end-stage renal disease receiving maintenance hemodialysis for at least 3 months. The cohort included all eligible outpatients treated at our institution during the study period (July 1, 2022 to January 20, 2024), and only sessions lasting at least 2 h were analyzed.

**Figure 1:**
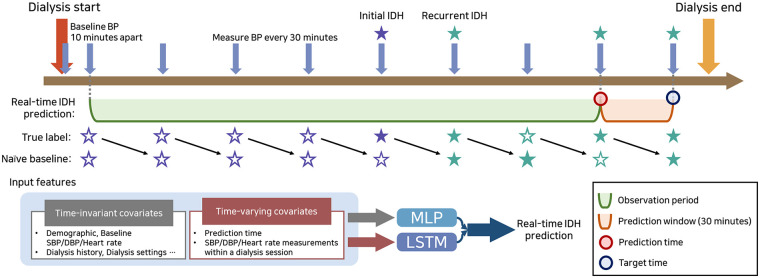
Schematic overview of real-time intradialytic hypotension (IDH) prediction. Input covariates are processed by a multi-layer perceptron (MLP) and a long short-term memory (LSTM) model. The naïve baseline uses the previous IDH occurrence to predict the next.

As part of a patient safety initiative, BP measurements were taken twice at baseline (10 min apart) and every 30 min during dialysis, an increase from the standard hourly measurements (Figure 1). All measurements were performed by trained nursing staff using the integrated oscillometric module of the GE CARESCAPE B155M monitor, on the upper arm contralateral to the vascular access site with the patient in the supine position.

These BP measurements were synchronized with real-time data from the therapy data monitoring system (Fresenius®) to improve IDH detection accuracy. The study adhered to the principles of the Declaration of Helsinki. No formal sample size calculation was performed; all available hemodialysis sessions from the 66 eligible patients were included. This study was approved by the Institutional Review Board of Seoul National University Bundang Hospital, which waived the requirement for informed consent (No. B-2210-789-103). This retrospective observational study was not prospectively registered.

### 2.2 Definitions of IDH and recurrent IDH

We used five IDH definitions:

1. Nadir90: nadir systolic BP (SBP) < 90 mm Hg
2. Nadir100: nadir SBP < 100 mm Hg
3. Fall20/MAP10: SBP decrease ≥20 mm Hg or mean arterial pressure (MAP) decrease ≥10 mm Hg from baseline
4. Fall20: SBP decrease ≥20 mm Hg from baseline
5. Fall30: SBP decrease ≥30 mm Hg from baseline.

These definitions were categorized into nadir-based (nadir90 and nadir100) and fall-based (Fall20/MAP10, Fall20, and Fall30). The results for each category are presented as the average outcome values from the definitions within that category. Recurrent IDH was defined as an event occurring at least 30 min after the initial IDH event, distinguishing it from a BP recheck after the initial event.

Analyses were conducted at two levels to assess the frequency of IDH. At the session level, we calculated the proportion of dialysis sessions with IDH by dividing the number of sessions with at least one IDH event by the total number of sessions. We calculated the proportion of IDH events at the BP measurement level by dividing the number of BP measurements meeting IDH criteria by the total number of BP measurements recorded during all dialysis sessions.

### 2.3 Naïve baseline for real-time IDH prediction

We developed a naïve baseline that predicts IDH events based solely on the most recent BP measurements to evaluate the impact of recurrence patterns on performance metrics. This simple rule-based model predicts the likelihood of IDH occurrence at the next BP measurement based on whether IDH was present in the current measurement that requires no training (Figure 1). Since this approach mirrors basic clinical practice, it serves as a benchmark for evaluating more complex real-time IDH prediction models. By design, this model predicted no IDH occurrence until the initial event was detected, resulting in a precision, recall, and F1 score of 0, with an AUROC of 0.5 for the initial event prediction.

### 2.4 Baseline model development

We developed real-time prediction models to predict IDH events 30 min before BP measurement (Figure 1). At each prediction point, vital-sign measurements (SBP, DBP, and heart rate) from the preceding 30-minute observation window are used as time-series input, and the model outputs an IDH occurrence probability for the next BP measurement. Following the conventional approach, baseline models predicted all IDH events during dialysis without distinguishing recurrence. Model performance was evaluated separately for initial and recurrent events.

The input features comprised (i) time-invariant features, including demographics, diagnosis history, prescription information, laboratory results, and dialysis information, and (ii) time-varying covariates, including elapsed time from the start of dialysis (i.e., time to predict) and vital signs (BP and heart rate) measured at 30-min intervals (Supplementary Table S1). Time-invariant features were processed with a multi-layer perceptron, and time-series data with a long short-term memory (LSTM) network (Figure 1). Outputs from both components were concatenated and passed through a linear layer for prediction.

The dataset was split into training and test sets (8:2 ratio) at the patient level with a five-fold cross-validation for hyperparameter tuning (Supplementary Materials S1, S2). The patient-level split was determined by selecting a random seed that yielded IDH prevalence rates within 1–2 percentage points between the training and test sets across all five IDH definitions. An ensemble of the five cross-validated models was used for testing. Model implementation utilized Python 3.10.10, PyTorch 2.0.0, and an NVIDIA RTX 4080.

To evaluate performance consistency across architectures, we additionally implemented PatchTST (24), TimesNet (25), ConvMixer (26), a temporal convolutional network (TCN) (27), and (v) an LSTM with an attention mechanism (LSTMWithAttention). These models were evaluated under three configurations: (1) baseline input only, (2) inclusion of a binary indicator of IDH occurrence, and (3) detailed IDH occurrence information.

### 2.5 Performance enhancement for individual IDH occurrence type

We implemented three different modeling strategies to improve the prediction accuracy for each type of IDH occurrence. First, we developed single-task models for each type of occurrence. Single-task models for the initial IDH were trained using the BP measurements collected up to the initial IDH occurrence in each dialysis session, while the model for recurrent events was trained using the data collected after the initial event. Second, we enhanced the prediction models by incorporating IDH occurrence information in two ways: (1) a binary indicator of whether IDH had occurred before the prediction point and (2) detailed IDH occurrence information, including the time elapsed since the initial and last occurrences, the total number of occurrences up to the prediction point, and SBP/diastolic blood pressure values at the initial and last IDH occurrences.

Finally, to optimize the model’s performance by balancing the focus between the initial and recurrent event predictions, we implemented a weighted loss function defined as

$$L_{total}=L_{target}+\alpha *L_{other}\;$$

where $L_{target}\;$ and $L_{other}$ represent the CrossEntropyLoss for the target occurrence (i.e., initial or recurrent) and the remaining type, respectively. The weighting factor α was tuned using values ranging from 0.01 to 0.9, and model selection was based on AUROC performance for the target IDH occurrence type.

All recurrence-related covariates are derived from events already observed within the current session at the prediction point. The prior-occurrence indicator, cumulative event count, elapsed time since the last IDH, and BP values at prior occurrences all take their defined null or zero values before the first IDH event in a session, and no future or contemporaneous information is used.

### 2.6 Model evaluation

The IDH prediction models were developed as binary classifiers and evaluated across multiple metrics: accuracy, precision, recall, F1 score, AUROC, area under the precision-recall curve (AUPRC), Matthews correlation coefficient, and negative predictive value. The AUROC was primarily reported as the main performance metric for model comparison.

To evaluate how recurrence patterns affected model performance across different IDH definitions and occurrence types, we analyzed the prediction score distributions, visualizing the risk distributions by overlaying the density plots of the prediction scores for IDH and non-IDH cases. These plots visually represent the model’s ability to distinguish between IDH and non-IDH cases, with larger non-overlapping areas indicating superior discriminative power, which corresponded to higher AUROC values (28).

### 2.7 Adversarial training

Adversarial training was motivated by the positive correlation between IDH occurrence and recurrence rates (Supplementary Figure S1), since, if left unconstrained, an occurrence-aware encoder may concentrate discriminative capacity in IDH-prone, high-recurrence patients, reducing performance consistency across pre-dialysis SBP subgroups.

The adversarial network comprised a two-layer multi-layer perceptron sharing the same hidden representations as the main prediction model, trained to perform binary classification of IDH recurrence status.

The adversarial loss was incorporated into the total loss function as follows:

$$L_{total}=L_{cls}-\;\lambda_{adv}*\;L_{adv}$$

where *L_cls_* represents the main classification loss, *L_adv_* denotes the adversarial loss, and *λ_adv_* is the adversarial weight parameter. The learning rate for the adversarial optimizer was set to one-tenth of that of the main model optimizer, with other training conditions remaining consistent. The *λ_adv_* was tuned from 1e-4 to 2e-5. To evaluate model robustness, we analyzed performance across sessions stratified by baseline systolic blood pressure (SBP), using 150 mmHg as the threshold, as the underlying mechanisms of IDH may differ between these groups.

### 2.8 Feature importance

To enhance model interpretability, we assessed the feature importance using the integrated gradient method (29) implemented using the Captum 0.7.0 package. Since the test inference was performed using an ensemble of five models from cross-validation, we averaged the feature importance values across these five models to obtain the final values. We scaled the sum of the feature importance values to a total of 100 for improved interpretability.

### 2.9 Statistical analysis

Given the variation in the number of dialysis sessions per patient, we reported the baseline characteristics of the study population using mean values across sessions for each patient (30). We employed t-tests for continuous variables and two-proportion z-tests for categorical variables to compare the covariate distribution between the training and test datasets.

The confidence intervals (95%) for the model performance metrics were calculated using the bootstrap method with 1000 iterations. The AUROC values between different models were compared using DeLong’s test. For feature importance, 95% confidence intervals were calculated using bootstrapping with 50 iterations of the integrated gradient calculation.

## 3 Results

### 3.1 Baseline characteristics

This study included 66 patients, categorized into training (*N* = 52) and test groups (*N* = 14; Table 1 and Supplementary Table S2). The mean (± standard deviation) age was 67.1 ± 15.7 years in the training group and 70.2 ± 10.8 years in the test group, with females accounting for 44.2% and 42.9% in each group, respectively. Baseline SBP was 144.7 ± 17.5 mm Hg in the training group and 149.9 ± 12.9 mm Hg in the test group. No significant differences in baseline characteristics were observed between the training and test groups. Both groups had long dialysis vintage (90.8 ± 57.5 months in the training group and 71.7 ± 41.6 months in the test group), severely reduced residual renal function (mean eGFR: 7.0 mL/min/1.73m² in the training group and 6.3 mL/min/1.73m² in the test group), and high antihypertensive medication use including ARBs and alpha-blockers (Table 1).

**Table 1:** Baseline characteristics of the study population.

Characteristic	Training (*N* = 52)	Test (*N* = 14)	*P*-value
Total session, n	10179	2588	
Session number per patient, n	195.8 (78.5)	185.9 (94.4)	0.723
Age, years^a^	67.1 (15.7)	70.2 (10.8)	0.398
Sex, female, *n* (%)	23 (44.2)	6 (42.9)	1.000
BMI, kg/m^2^^a^	22.5 (2.7)	23.5 (3.2)	0.298
Baseline vital measurements^a^			
Systolic BP, mmHg	144.7 (17.5)	149.9 (12.9)	0.228
Diastolic BP, mmHg	65.5 (11.7)	68.7 (10.4)	0.331
Heart rate, bpm	72.5 (9.4)	71.2 (10.3)	0.674
Dialysis cause, %^a^			
Diabetes mellitus	23.1 (42.1)	14.3 (35.0)	0.425
Hypertension	15.4 (36.1)	28.6 (45.2)	0.313
Dialysis settings^a^			
Dry weight, kg	57.8 (9.6)	60.1 (12.7)	0.536
Inter-dialytic weight change, kg	1.7 (1.1)	2.1 (0.6)	0.078
Dialysis adequacy			
spKt/V, (unitless)	1.8 (0.3)	1.8 (0.3)	1.000
eKt/V, (unitless)	1.6 (0.2)	1.5 (0.2)	0.112
nPCR, g/kg/day	0.8 (0.1)	0.8 (0.2)	1.000
Residual renal function, %	7.0 (12.0)	13.2 (26.7)	0.397
Dialysis vintage, month	90.8 (57.5)	71.7 (41.6)	0.174
Diagnosis, %^a^			
AKI	13.5 (34.1)	21.0 (41.0)	0.530
Cancer	19.6 (39.3)	42.0 (49.5)	0.117
Diabetes mellitus	42.9 (49.1)	35.7 (47.9)	0.620
Prescription within 30 days, %^a^			
Antihypertensive			
Alpha blockers	53.7 (42.1)	40.5 (42.6)	0.302
Beta blockers	42.1 (44.3)	22.9 (37.6)	0.103
ARBs	62.4 (39.9)	55.2 (45.0)	0.587
Diuretics	37.3 (43.9)	44.8 (44.1)	0.572
Laboratory test results^a^			
eGFR, mL/min/1.73m^2^	7.0 (3.3)	6.3 (1.2)	0.215
Creatinine, mg/dL	8.1 (2.4)	8.0 (1.6)	0.855
BUN, mg/dL	17.3 (7.6)	21.6 (18.4)	0.407

BMI, body mass index; BP, blood pressure; bpm, beats per minute; spKt/V, single-pooled Kt/V; eKt/V, Equilibrated Kt/V; nPCR, normalized protein catabolic rate; URR, urea reduction ratio; AKI, acute kidney injury; ARBs, angiotensin II receptor blockers; CCBs, calcium channel blockers; eGFR, estimated glomerular filtration rate; BUN, blood urea nitrogen.

a The statistics are derived by calculating the average values per session for each patient, then computing the overall mean and standard deviation of these averages to ensure balanced representation of all patients.

### 3.2 IDH recurrence patterns

**Figure 2:**
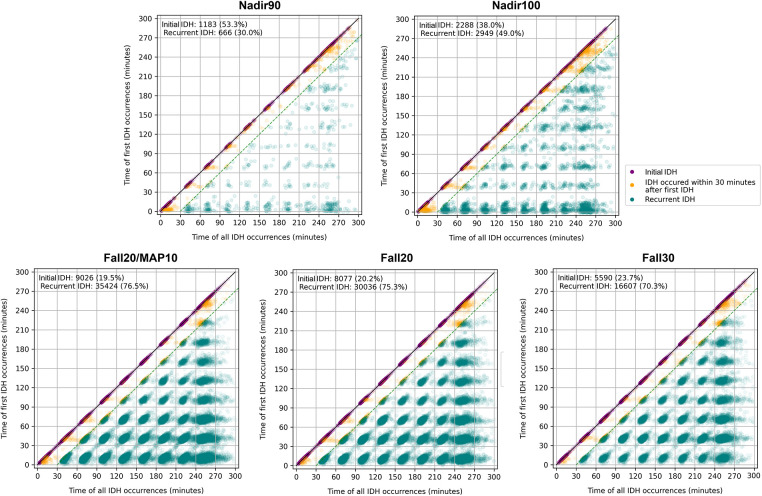
Recurrence pattern of intradialytic hypotension (IDH) during a dialysis session across different IDH definitions. Initial IDH (purple), recurrent IDH (green), and IDH within 30 min after the initial event (yellow) are shown. Percentages of initial and recurrent IDH occurrences are displayed.

Analysis of 12,767 dialysis sessions revealed distinct recurrence patterns of IDH following the initial IDH event (Figure 2 and Table 2). The frequency of IDH occurrence measurements meeting the IDH criteria ranged from 0.7–10.4% before the initial IDH event, increasing to 11.7–65.7% after the initial event across the five IDH definitions (*p* < 0.001 for all definitions). Recurrent IDH was observed in 14.3–63.3% of sessions with IDH occurrence. The session-level prevalence of IDH varied by definition as follows: 3.3% for Nadir90, 8.7% for Nadir100, 58.0% for Fall20/MAP10, 51.2% for Fall20, and 32.4% for Fall30. Per-patient IDH occurrence rate and recurrence rate were positively associated across all five IDH definitions, with IDH-prone patients tending to experience more intra-session recurrences (Supplementary Figure S1).

**Table 2:** Prevalence and recurrence patterns of intradialytic hypotension (IDH).

IDH prevalence	IDH definitions				
	Nadir90	Nadir100	Fall20/MAP10	Fall20	Fall30
Session-level, %					
All IDH occurrences	3.3	8.7	58.0	51.2	32.4
Recurrence in sessions with IDH	14.3	24.2	63.3	60.8	51.3
BP measurement-level, %					
All IDH occurrences	1.4	4.6	39.2	34.2	20.5
Before initial IDH^a^	0.7	1.6	10.4	8.8	5.6
After initial IDH	11.7	29.2	65.7	65.4	61.5
Average reoccurrence count in sessions with IDH, n	0.9	1.6	4.1	3.9	3.2

IDH, intradialytic hypotension; SBP, systolic blood pressure; Fall20/MAP10, kidney disease outcomes quality initiative; Nadir90, SBP < 90 mm Hg; Nadir100, SBP < 100 mm Hg; Fall20, SBP drop of 20 mm Hg; Fall30, SBP drop of 30 mm Hg, BP, blood pressure.

a This includes all measurements from the initiation of the session up to and including the initial IDH event. A significant difference in IDH prevalence was observed between the periods before and after the initial IDH occurrence across all IDH definitions (*p* < 0.001).

### 3.3 Performance of baseline models and naïve baseline

The baseline models trained to predict all IDH occurrences showed significant performance differences in predicting initial vs. recurrent IDH events across various IDH definitions (Table 3 and Supplementary Tables S3–S7). Both nadir-based and fall-based definitions revealed significant differences in accuracy, F1 score, AUROC, and AUPRC between the initial and recurrent IDH predictions (*p* < 0.001), except for AUROC in the fall-based definitions. The performance gaps were particularly notable in the F1 score and AUPRC for fall-based definitions, with differences of approximately 0.4.

**Table 3:** Performance comparison of naïve baseline and baseline models across different intradialytic hypotension (IDH) definitions and occurrence types. Performance metrics are presented as point estimates with 95% confidence intervals.

IDH definitions	Model types	IDH occurrence types	Performance metrics			
			Accuracy	F1	AUROC	AUPRC
Nadir-based^a^	Naïve baseline	All IDH	0.964 [0.961, 0.967]	0.056 [0.036, 0.082]	0.516 [0.507, 0.528]	0.028 [0.025, 0.032]
		Initial IDH^b^	**0.990 [0.988, 0.992]**	0.000 [0.000, 0.000]	0.500 [0.500, 0.500]	0.010 [0.009, 0.012]
		Recurrent IDH	0.599 [0.570, 0.631]	**0.086 [0.055, 0.126]**	**0.590 [0.552, 0.620]**	**0.159 [0.138, 0.179]**
		Difference^c^	−0.391 [−0.422, −0.360]	0.086 [0.055, 0.117]	0.090 [0.052, 0.128]	0.149 [0.128, 0.170]
	Baseline model	All IDH	0.966 [0.963, 0.969]	0.408 [0.373, 0.444]	**0.956 [0.947, 0.964]**	0.394 [0.353, 0.434]
		Initial IDH	**0.979 [0.977, 0.982]**	0.312 [0.260, 0.364]	0.953 [0.939, 0.964]	0.282 [0.233, 0.335]
		Recurrent IDH	0.762 [0.730, 0.792]	**0.436 [0.377, 0.493]**	0.824 [0.786, 0.859]	**0.467 [0.395, 0.550]**
		Difference^c^	−0.217 [−0.249, −0.185]	0.124 [0.045, 0.203]	−0.129 [−0.170, −0.088]	0.185 [0.101, 0.269]
Fall-based^a^	Naïve baseline	All IDH	0.842 [0.837, 0.848]	0.726 [0.716, 0.736]	**0.798 [0.792, 0.805]**	0.621 [0.608, 0.632]
		Initial IDH^b^	**0.928 [0.924, 0.934]**	0.000 [0.000, 0.000]	0.500 [0.500, 0.500]	0.072 [0.066, 0.076]
		Recurrent IDH	0.730 [0.720, 0.739]	**0.790 [0.781, 0.798]**	0.698 [0.687, 0.708]	**0.735 [0.723, 0.746]**
		Difference^c^	−0.198 [−0.209, −0.187]	0.790 [0.781, 0.799]	0.198 [0.187, 0.209]	0.663 [0.650, 0.676]
	Baseline model	All IDH	0.872 [0.867, 0.877]	0.791 [0.781, 0.800]	**0.937 [0.933, 0.941]**	0.875 [0.867, 0.883]
		Initial IDH	**0.923 [0.918, 0.929]**	0.453 [0.417, 0.488]	0.882 [0.869, 0.895]	0.480 [0.442, 0.518]
		Recurrent IDH	0.794 [0.784, 0.804]	**0.842 [0.834, 0.851]**	0.875 [0.867, 0.883]	**0.918 [0.911, 0.925]**
		Difference^c^	−0.129 [−0.141, −0.117]	0.389 [0.351, 0.427]	−0.007 [−0.024, 0.010]	0.438 [0.398, 0.478]

AUROC, area under the receiver operating characteristics curve; AUPRC, area under the precision-recall curve.

Bold values indicate the best-performing metric, and underlined values the second-best, within each IDH definition category and model type.

a Nadir-based definitions include Nadir90 (nadir systolic blood pressure (SBP) less than 90 mm Hg) and Nadir100 (nadir SBP less than 100 mm Hg). Fall-based definitions include Fall20/MAP10 (SBP decrease of 20 mm Hg or more or mean arterial pressure (MAP) decrease of 10 mm Hg or more from baseline), Fall20 (SBP decrease of 20 mm Hg or more from baseline), and Fall30 (SBP decrease of 30 mm Hg or more from baseline).

b By definition of the naïve baseline, F1 score and AUROC are 0.0 and 0.5 for predicting the initial IDH, respectively.

c P-values for the comparison between initial IDH and recurrent IDH performance are <0.001 for all metrics and model types, except for the AUROC of fall-based baseline model (*p* = 0.180).

**Figure 3:**
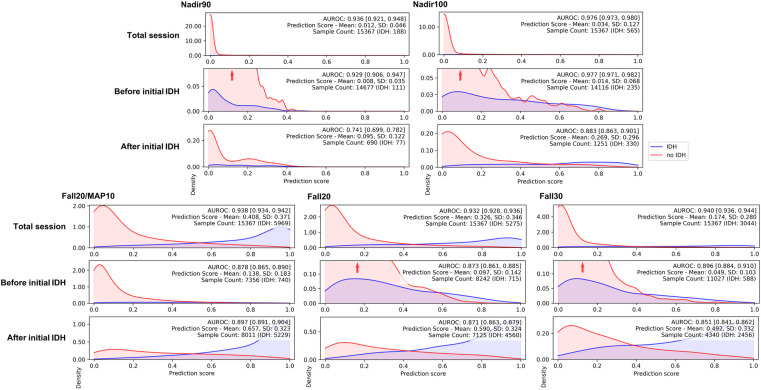
Prediction score distributions for intradialytic hypotension (IDH) and non-IDH cases across different IDH definitions and occurrence types. Blue and red lines represent the prediction score distributions for IDH and non-IDH cases, respectively. Greater separation between these distributions, indicated by larger non-overlapping areas, suggests improvements in the model’s discriminative ability and yields higher AUROC values. In the initial IDH prediction settings indicated by red arrows, the non-IDH density shows pronounced peaks. For the Nadir90 and Nadir100 definitions, these peaks exceed a value of 10, while for Fall20 and Fall30, they surpass 2 and 4, respectively.

The AUROC values for total dialysis sessions were higher than those for individual IDH occurrences across different IDH definitions and model types (Table 3), except for the naïve baseline in the nadir-based definitions. Additionally, the prediction score distributions showed marked differences before and after the initial IDH occurrence (Figure 3). The mean prediction scores increased from a range of 0.008–0.138 before the initial IDH to 0.095–0.657 after the initial IDH occurrence across all definitions, with a higher proportion of IDH cases after the initial IDH occurrence. These differences in prediction score distributions were most significant in fall-based definitions, where the elevation in the AUROC for all IDH occurrences compared with individual occurrence types was also the most notable.

The naïve baseline also showed significantly higher AUROC values for all IDH occurrences than the individual AUROC values for predicting initial and recurrent IDH in the fall-based definitions. Notably, even without any training process, the naïve baseline achieved an AUROC of 0.798 for all IDH occurrences in the fall-based definitions (Table 3).

### 3.4 Enhancing model performance by IDH occurrence type

**Figure 4:**
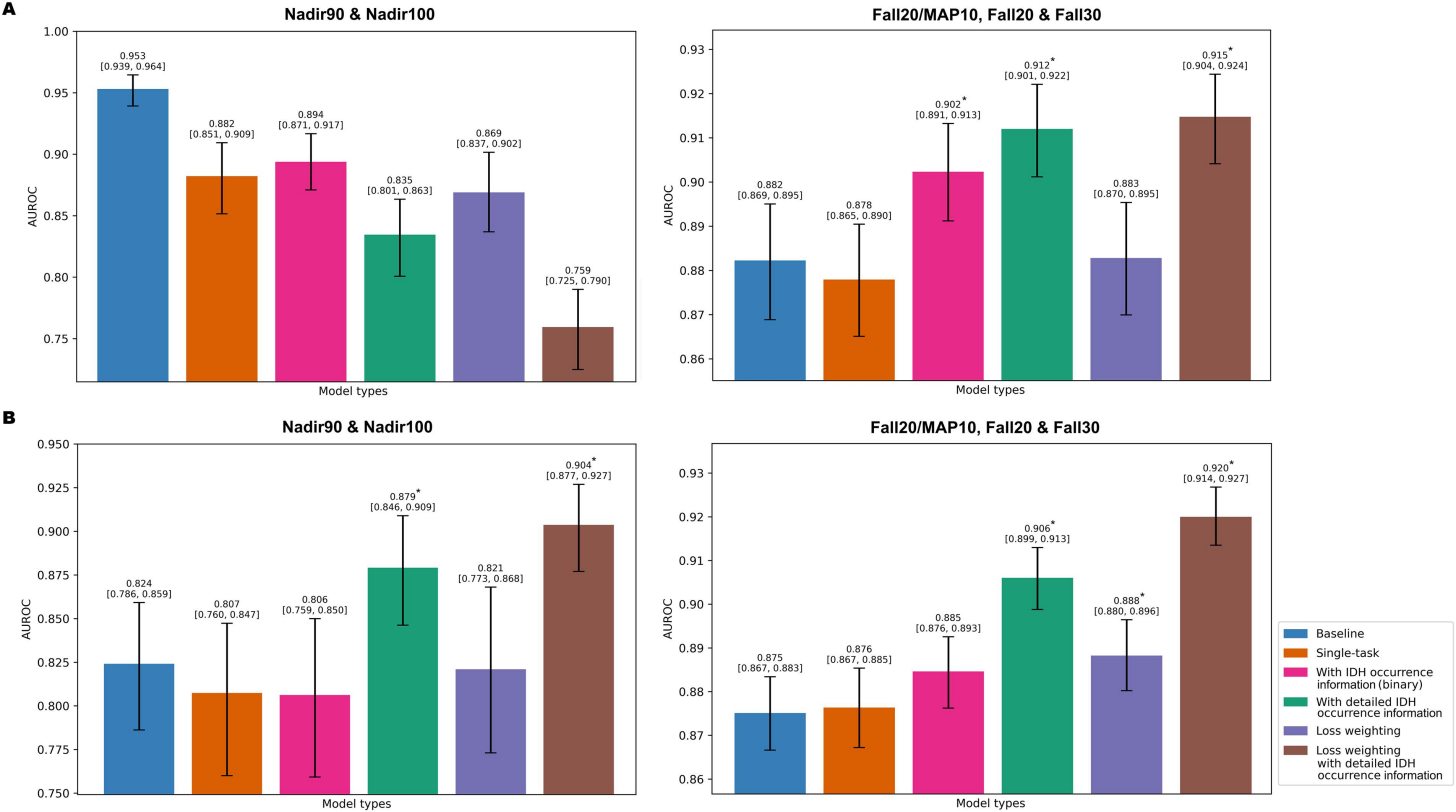
Performance enhancement of real-time intradialytic hypotension (IDH) prediction models on different IDH definitions and occurrence types. **(A)** initial IDH and **(B)** recurrent IDH. Error bars represent 95% confidence intervals. * indicates significant improvement in the model performance (*p* < 0.05) from the baseline model.

We observed significant performance improvements when developing real-time IDH prediction models specific to IDH occurrence types (Figure 4 and Supplementary Tables S3–S7). Providing detailed IDH occurrence information as input covariates resulted in AUROC improvements of 3.0 to 5.5 percentage points compared to the baseline model, including whether an earlier occurrence of an IDH event in the same dialysis session led to a 2 percentage point improvement for initial events using fall-based definitions.

Although neither single-task models nor loss weighting alone yielded significant improvements, the combination of loss weighting with detailed IDH occurrence information produced additional enhancements, achieving a maximum improvement of 8.0 percentage points for recurrent IDH events in the nadir-based definitions (Figure 4). However, these modeling approaches proved less effective for the initial IDH events in nadir-based definitions, resulting in performance below that of the baseline model.

**Figure 5:**
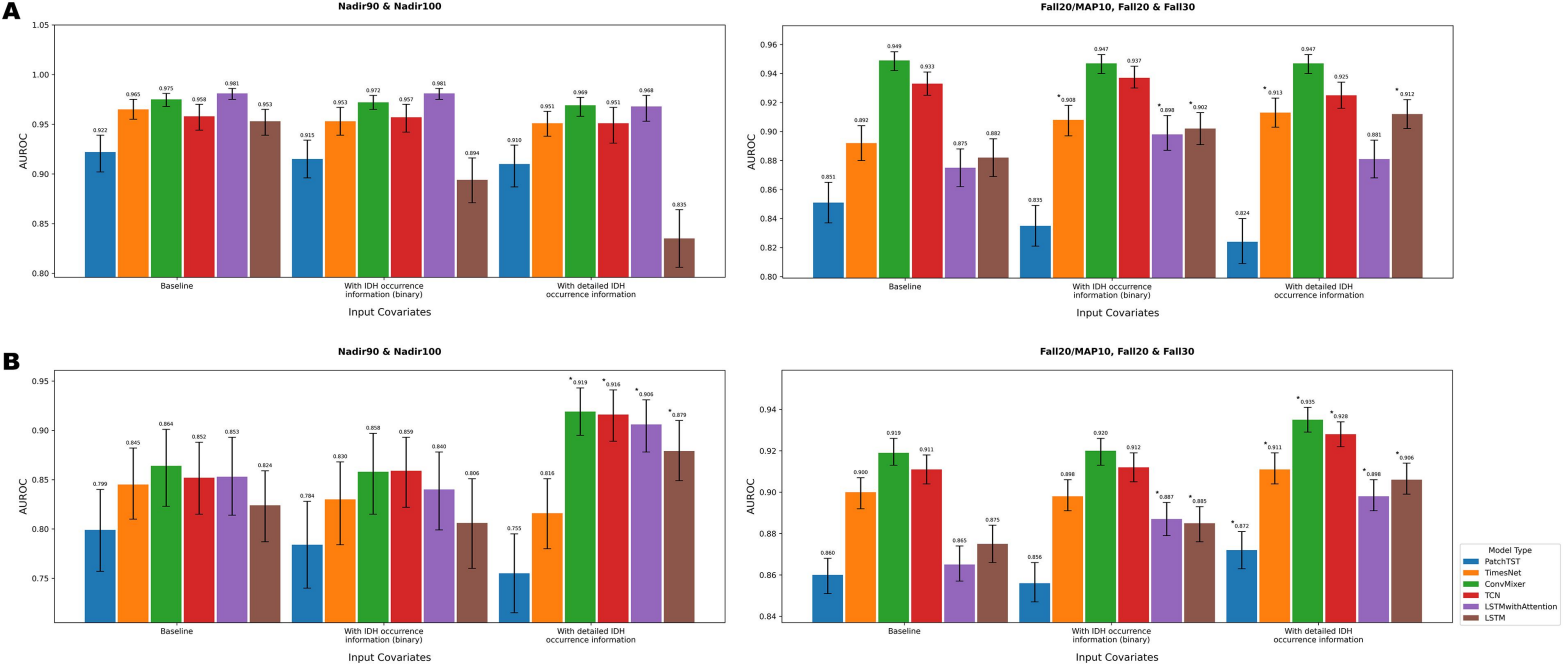
Performance comparison of real-time intradialytic hypotension (IDH) prediction models across different neural network architectures and input covariates. **(A)** Initial IDH and **(B)** recurrent IDH prediction performance across PatchTST, TimesNet, ConvMixer, TCN, LSTM with attention, and standard LSTM models. For each model, performance is compared under three input configurations: baseline input only, inclusion of binary IDH occurrence information, and inclusion of detailed IDH occurrence information. Error bars indicate 95% confidence intervals. * denotes a statistically significant improvement (*p* < 0.05) over the baseline input model within each neural network architecture.

Additionally, these trends were consistent across alternative deep learning architectures (Figure 5). ConvMixer and TCN consistently outperformed other models in both AUROC level and stability, particularly when incorporating detailed IDH occurrence information. For recurrent IDH events, convolution-based models achieved AUROC values exceeding 0.90 even in nadir-based definitions, with ConvMixer reaching up to 0.919 and TCN 0.916 (Figure 5). Fall-based definitions showed similarly high performance, with ConvMixer and TCN surpassing 0.93 AUROC. The inclusion of detailed recurrence information not only improved absolute performance but also narrowed the performance variability across models, suggesting that recurrence-aware feature design benefits both architecture robustness and generalization across IDH definitions.

A leave-one-subject-out sensitivity analysis confirmed that no single test patient disproportionately influenced the reported estimates across all model conditions (mean AUROC range 0.025; Supplementary Table S8).

### 3.5 Impact of adversarial training on model robustness

We evaluated model robustness across patient subgroups stratified by baseline SBP (Supplementary Table S9). While incorporating IDH occurrence information improved overall model performance, it led to increased performance disparities between patient subgroups, particularly in fall-based definitions (absolute difference in AUROC: 0.231, *p* < 0.001; Table 4). Adversarial training effectively mitigated these disparities, reducing the performance gap between higher and lower baseline SBP groups by 0.063 percentage points (*p* < 0.001) in fall-based definitions while maintaining strong overall performance [AUROC: 0.895 (0.884, 0.907)]. Similar trends were observed in nadir-based definitions, where adversarial training reduced the performance difference between subgroups from 0.038 to 0.004, albeit with a slight decrease in overall AUROC (0.879–0.860).

**Table 4:** Impact of adversarial training and intradialytic hypotension (IDH) occurrence features on model performance across different IDH definitions.

IDH Definitions	Performance (AUROC)				
Model Types	Total	Higher baseline SBP	Lower baseline SBP	Absolute *Δ*	*Δ* of *Δ* (+ Adv.)
Nadir-based					
Baseline	0.824 [0.786, 0.859]	0.754 [0.665, 0.837]	0.843 [0.803, 0.879]	0.089	−0.083
+ Adv.	0.787 [0.732, 0.836]	0.805 [0.730, 0.874]	0.799 [0.732, 0.860]	0.006	
With IDH occurrence features	0.879 [0.846, 0.909]	0.850 [0.774, 0.918]	0.888 [0.853, 0.919]	0.038	−0.034
+ Adv.	0.860 [0.825, 0.891]	0.857 [0.801, 0.910]	0.861 [0.822, 0.898]	0.004	
Fall-based					
Baseline	0.882 [0.869, 0.895]	0.871 [0.861, 0.880]	0.836 [0.813, 0.859]	0.035*	−0.002
+ Adv.	0.865 [0.856, 0.874]	0.863 [0.852, 0.873]	0.830 [0.807, 0.852]	0.033*	
With IDH occurrence features	0.912 [0.901, 0.922]	0.888 [0.879, 0.897]	0.657 [0.643, 0.671]	0.231**	−0.063**
+ Adv.	0.895 [0.884, 0.907]	0.877 [0.867, 0.887]	0.709 [0.687, 0.732]	0.168**	

Performance metrics are presented as point estimates with 95% confidence intervals.

IDH, intradialytic hypotension; SBP, systolic blood pressure; Adv., adversarial training.

* *p* < 0.01.

** *p* < 0.001.

### 3.6 Calibration and threshold operating characteristics

Both the baseline model and the model with detailed IDH occurrence information were well calibrated across all five IDH definitions without a *post-hoc* calibration step (Brier scores: 0.007–0.160; Supplementary Figure S2).

At the F1-maximizing threshold, PPV varied substantially across IDH definitions for Initial IDH sessions, ranging from 0.129 (Nadir90, alert rate 1.6%) to 0.507 (Fall20/MAP10, alert rate 10.3%; Supplementary Figure S3). For Recurrent IDH sessions, PPV was higher (exceeding 0.81 for Fall20/MAP10), but this largely reflects the higher event prevalence in that stratum rather than superior discriminative performance. These patterns highlight that both PPV and alert rate must be jointly considered when selecting an operating threshold (Supplementary Figure S3).

### 3.7 Feature importance

**Figure 6:**
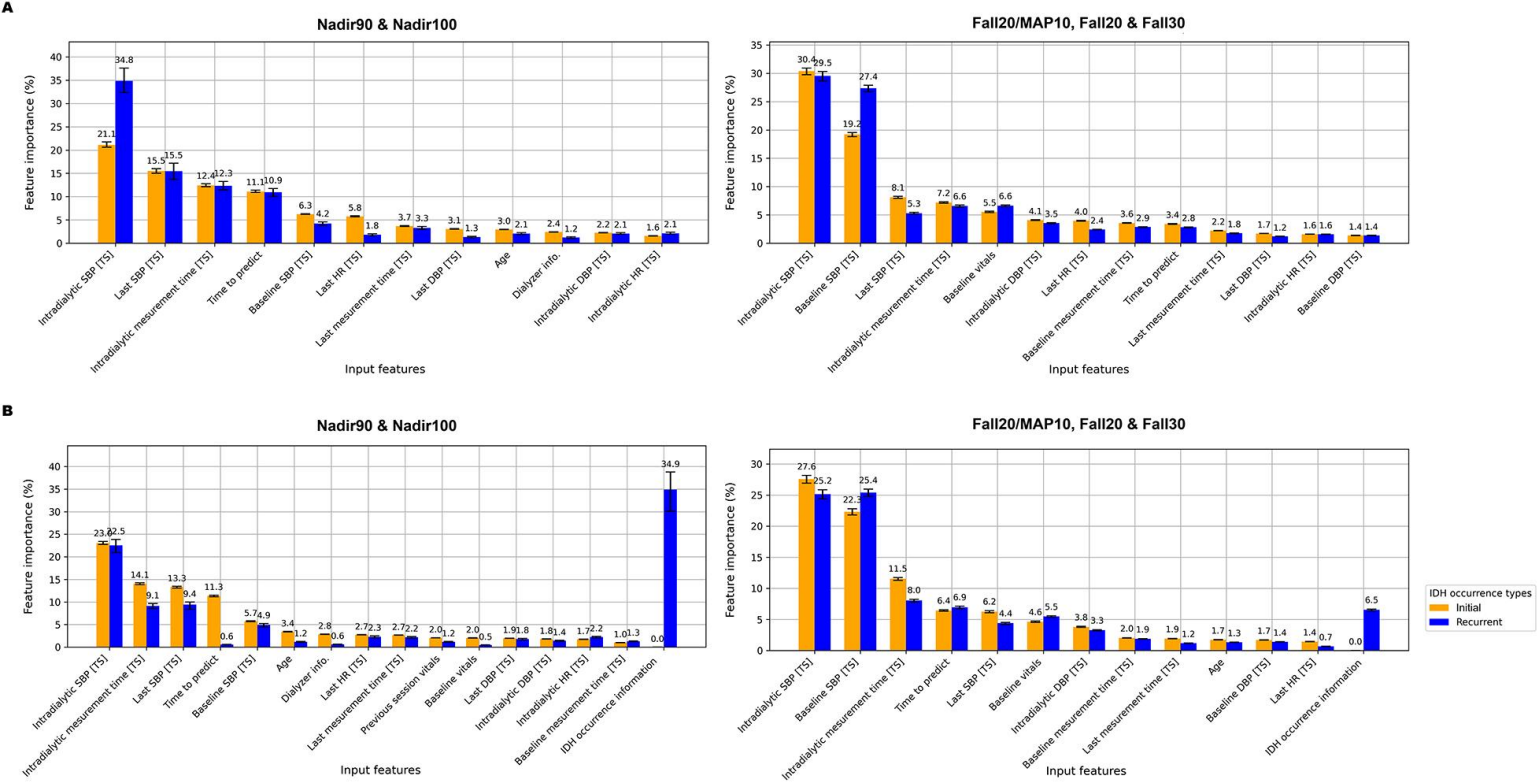
Feature importance across different intradialytic hypotension (IDH) definitions and occurrence types. **(A)** shows the feature importance of the baseline model, while **(B)** presents the feature importance of the model incorporating IDH occurrence features. Error bars represent 95% confidence intervals. [TS] indicates time-series vital data.

Analysis of feature importance demonstrated that the real-time IDH prediction model primarily relied on time-series data, with utilization patterns varying across IDH definitions (Figure 6). Intradialytic SBP provided as time-series data emerged as the most influential variable across all model types, IDH definitions, and occurrence types, with normalized importance values ranging from 21.1 to 34.8. For nadir-based definitions, the last SBP was ranked as the second most important feature, while the baseline SBP held this position in the fall-based definitions. Measurement time consistently appeared among the top three influential variables.

The incorporation of detailed IDH occurrence information markedly shifted the overall feature importance distribution (Figure 6B). In recurrent IDH predictions, IDH occurrence features proved to be highly influential and have frequently emerged as primary predictors. Notably, in the nadir-based definitions of recurrent events, these features were the most important variables, with a feature importance of 34.9. The addition of IDH occurrence information generally diminished the relative importance of time-series vital-sign data, particularly in the context of recurrent events.

## 4 Discussion

This study revealed that the conventional approach to developing and evaluating real-time IDH prediction models should consider the distinction between types of IDH occurrence. Treating all IDH occurrences during a dialysis session as equivalent can lead to overestimation of the model performance and misinterpretation of its clinical value, particularly in settings with strong recurrence patterns.

We found substantial and consistent performance gaps between predicting initial and recurrent IDH events across all IDH definitions and metrics (Table 3). For fall-based definitions, the baseline model showed differences of approximately 0.4 in both the F1 score and AUPRC. These gaps are largely attributed to the higher proportion of positive cases when predicting recurrent events, which reflects the intrinsic recurrence patterns of IDH. Without separating occurrence types, performance evaluation may be biased toward these higher-prevalence recurrent events, giving an inaccurate impression of real-world clinical utility.

The distinct physiological and environmental mechanisms underlying initial and recurrent events further justify their separate evaluation. Initial IDH typically occurs in patients who are hemodynamically unstable at dialysis onset, reflecting an inability to maintain BP homeostasis in response to ultrafiltration-induced fluid volume changes (31). In contrast, recurrent IDH occurs in the context of a more compromised post-event hemodynamic state, the effectiveness and timing of interventions, and possible psychological stress from prior symptoms (32). These differing etiologies imply that the same prediction model is unlikely to perform equally well for both types of events.

We also observed that the AUROC values calculated across all IDH events were consistently higher than those for initial or recurrent events alone (Table 3). Notably, even the naïve baseline, which relied solely on the most recent IDH occurrence status, achieved AUROC ranging from 0.510 to 0.802 across different definitions (Table 3 and Supplementary Tables S3–S7). Although simplistic, this approach inherently leverages recurrence patterns and serves as a practical reference point for fairer comparisons across datasets with varying IDH prevalence and recurrence tendencies.

Given these recurrence patterns, it is not surprising that training on all events caused IDH occurrence status to become a dominant predictor. This was evident both in the substantial performance improvements when occurrence information was explicitly provided (Figures 4, 5) and in feature importance analyses showing its primary role in predicting recurrent events (Figure 6B). While time-series vital signs inherently contain this information, explicitly including it as a covariate may help the model focus on relevant temporal patterns rather than inferring occurrence status indirectly. The performance gain in initial-event sessions likely reflects representation-level regularization, where an explicit null prior may redirect the encoder toward complementary hemodynamic features, aided by joint training with recurrent sessions. Beyond the evaluation implications described above, we also examined whether these recurrence patterns could be actively leveraged to improve predictive modeling.

Despite the differing mechanisms between the initial and recurrent IDH events, a single integrated model for all IDH events remains an effective modeling strategy for real-time IDH prediction. Single-task models dedicated to each event type did not improve performance (Figure 4), likely due to reduced training data and the intrinsic relationship between the two prediction tasks. Therefore, we applied loss weighting to balance the importance of predicting both occurrence types. When combined with detailed IDH occurrence information, this approach improved AUROC by 3.3–8.0 percentage points over the baseline, underscoring that while evaluation should distinguish occurrence types, integrating modeling can achieve optimal predictive performance.

However, these strategies may compromise robustness and performance consistency. Models leveraging recurrence patterns showed substantial performance disparities between patient subgroups stratified by baseline SBP (Table 4). Adversarial training effectively mitigated these disparities while maintaining overall performance, illustrating the trade-off between exploiting recurrence patterns for higher accuracy and preserving stable performance across a diverse patient population.

Based on these findings, we propose three recommendations for real-time IDH prediction model development and evaluation: (1) evaluate models separately for initial and recurrent events rather than aggregating all events; (2) report the performance of a naïve baseline model to provide a meaningful benchmark and enable fairer cross-study comparison; and (3) report IDH prevalence and recurrence patterns within the dataset to contextualize performance metrics.

Furthermore, the analytical framework proposed here is broadly applicable to clinical time-series problems in which an initial event shifts the patient’s physiological baseline and elevates recurrence risk. Such dynamics are common across clinical domains, including recurrent myocardial infarction, ischemic stroke (33), and other conditions with recognized event clustering (34). In each case, modeling that explicitly accounts for recurrence history is needed both to avoid evaluation bias from pooling occurrence types and to capture history-dependent predictive signals. Similar considerations apply to hypoglycemia prediction during hemodialysis using continuous glucose monitoring, where an initial episode alters the patient’s vulnerability and motivates recurrence-specific modeling (35).

This study had several limitations. First, the single-center dataset of 66 patients may limit generalizability. Nevertheless, our core finding that intra-session IDH recurrence systematically inflates aggregate performance metrics is unlikely to be center-specific, as it is grounded in well-established hemodynamic mechanisms—progressive volume depletion and exhausted vasomotor reserve (32, 36, 37)—common to all hemodialysis settings. Furthermore, IDH occurrence rate and intra-session recurrence rate were positively associated across all five definitions (Supplementary Figure S1); given that IDH prevalence rates in our cohort were comparable to or lower than those in previously published cohorts, a similar degree of intra-session recurrence would be expected in other hemodialysis populations.

Second, we could not improve initial-event prediction for nadir-based definitions, likely due to the limited post-event samples from their lower overall prevalence (Figure 3 and Table 2). Third, incorporating intervention data from dialysis sessions may further enhance recurrent-event prediction. Fourth, because multiple sessions were recorded per patient, the session-level confidence intervals and significance tests may be overly narrow.

Although this study is the first to report IDH recurrence patterns, such patterns are unlikely to be unique to our dataset. The recurrence pattern is clinically plausible, and IDH prevalence at both the session and BP measurement levels (Table 2) was comparable to or lower than previous reports (9, 17), indicating that our cohort did not have unusually poor hemodynamic stability. Nonetheless, validating recurrence patterns in other clinical settings remains necessary.

In conclusion, this study shows that distinguishing initial and recurrent IDH events is essential for accurate model development and evaluation, as aggregating all events overestimates performance and masks clinically meaningful differences. By explicitly modeling recurrence patterns and incorporating detailed occurrence information, we improved the AUROC by up to 8.0 percentage points and narrowed performance variability across architectures. These results indicate that recurrence patterns are not only a source of bias in evaluation but also a consistent modeling signal that, when leveraged appropriately, enhances both accuracy and robustness. We recommend that future studies report recurrence statistics, include naïve baselines, separately evaluate initial and recurrent events, and adopt recurrence-aware modeling strategies to improve clinical applicability and reliability of real-time IDH prediction.

## Data Availability

The datasets presented in this article are not available for public sharing due to restrictions regarding internal patient privacy. Requests to access the datasets should be directed to Hyung-Jin Yoon, hjyoon@snu.ac.kr. The code for the analysis and model is available on GitHub (https://github.com/SiunKim/IDH-Recurrence-Prediction).

## References

[1] ReevesPB Mc CauslandFR. Mechanisms, clinical implications, and treatment of intradialytic hypotension. Clin J Am Soc Nephrol. (2018) 13:1297–303. 10.2215/CJN.1214101729483138 PMC6086712

[2] WangJ YaoJ ZhuX WangT LuJ WeiQ. Impact of frequent intradialytic hypotension on quality of life in patients undergoing hemodialysis. BMC Nephrol. (2023) 24:209. 10.1186/s12882-023-03263-637452301 PMC10347841

[3] Tislér A Akócsi K Borbás B Fazakas L Ferenczi S Görögh S. The effect of frequent or occasional dialysis-associated hypotension on survival of patients on maintenance haemodialysis. Nephrol Dial Transplant. (2003) 18:2601–5. 10.1093/ndt/gfg450 14605284

[4] ChouJA Kalantar-ZadehK MathewAT. A brief review of intradialytic hypotension with a focus on survival. Semin Dial. (2017) 30:473–80. 10.1111/sdi.1262728661565 PMC5738929

[5] ParkYW YunD YuY KimSH ParkS KimYC. Intradialytic hypotension and worse outcomes in patients with acute kidney injury requiring intermittent hemodialysis. Kidney Res Clin Pract. (2024) 45:77–85. 10.23876/j.krcp.23.18838389146 PMC12824503

[6] ChouJA StrejaE NguyenDV RheeCM ObiY InrigJK. Intradialytic hypotension, blood pressure changes and mortality risk in incident hemodialysis patients. Nephrol Dial Transplant. (2018) 33:149–59. 10.1093/ndt/gfx03728444336 PMC5837776

[7] InrigJK. Beware intradialytic hypotension: how low is too low? Clin J Am Soc Nephrol. (2018) 13:1453–4. 10.2215/CJN.1015081830237217 PMC6218818

[8] KuipersJ VerboomLM IpemaKJR PaansW KrijnenWP GaillardCAJM. The prevalence of intradialytic hypotension in patients on conventional hemodialysis: a systematic review with meta-analysis. Am J Nephrol. (2019) 49:497–506. 10.1159/00050087731129661 PMC6604263

[9] Arcentales-VeraK Vera-MendozaMF Cevallos-SalasC Garcia-AguileraMF Fuenmayor-GonzalezL. Prevalence of cardiovascular instability during hemodialysis therapy in hospitalized patients: a systematic review and meta-analysis. Sci Prog. (2024) 107(4):00368504241308982. 10.1177/0036850424130898239726219 PMC11686621

[10] Benaroia M Iliescu EA. Oral intake during hemodialysis: is there an association with intradialytic hypotension? Hemodial Int. (2008) 12:62–5. 10.1111/j.1542-4758.2008.00242.x 18271843

[11] Song JH Park GH Lee SY Lee SW Lee SW Kim MJ. Effect of sodium balance and the combination of ultrafiltration profile during sodium profiling hemodialysis on the maintenance of the quality of dialysis and sodium and fluid balances. J Am Soc Nephrol. (2005) 16:237–46. 10.1681/ASN.2004070581 15563561

[12] DubinR OwensC GasperW GanzP JohansenK. Associations of endothelial dysfunction and arterial stiffness with intradialytic hypotension and hypertension. Hemodial Int. (2011) 15:350–8. 10.1111/j.1542-4758.2011.00560.x21658174 PMC3143472

[13] K/DOQI Workgroup. K/DOQI clinical practice guidelines for cardiovascular disease in dialysis patients. Am J Kidney Dis. (2005) 45(Supplement 3):S1–153. 10.1053/j.ajkd.2005.01.019 15806502

[14] Eknoyan G Beck GJ Cheung AK Daugirdas JT Greene T Kusek JW. Effect of dialysis dose and membrane flux in maintenance hemodialysis. N Engl J Med. (2002) 347:2010–9. 10.1056/NEJMoa021583 12490682

[15] Dong J Wang K He J Guo Q Min H Tang D. Machine learning-based intradialytic hypotension prediction of patients undergoing hemodialysis: a multicenter retrospective study. Comput Methods Programs Biomed. (2023) 240:107698. 10.1016/j.cmpb.2023.107698 37429246

[16] Lee H Moon SJ Kim SW Min JW Park HS Yoon HE. Prediction of intradialytic hypotension using pre-dialysis features-a deep learning–based artificial intelligence model. Nephrol Dial Transplant. (2023) 38:2310–20. 10.1093/ndt/gfad064 37019834

[17] LeeH YunD YooJ YooK KimYC KimDK. Deep learning model for real-time prediction of intradialytic hypotension. Clin J Am Soc Nephrol. (2021) 16:396–406. 10.2215/CJN.0928062033574056 PMC8011016

[18] Lin CJ Chen CY Wu PC Pan CF Shih HM Huang MY. Intelligent system to predict intradialytic hypotension in chronic hemodialysis. J Formos Med Assoc. (2018) 117:888–93. 10.1016/j.jfma.2018.05.023 29941330

[19] YunD YangHL KimSG KimK KimDK OhKH. Real-time dual prediction of intradialytic hypotension and hypertension using an explainable deep learning model. Sci Rep. (2023) 13:18054. 10.1038/s41598-023-45282-137872390 PMC10593747

[20] Kim HW Heo SJ Kim M Lee J Park KH Lee G. Deep learning model for predicting intradialytic hypotension without privacy infringement: a retrospective two-center study. Front Med. (2022) 9:878858. 10.3389/fmed.2022.878858 PMC9300869 35872786

[21] Chen JB Wu KC Moi SH Chuang LY Yang CH. Deep learning for intradialytic hypotension prediction in hemodialysis patients. IEEE Access. (2020) 8:82382–90. 10.1109/ACCESS.2020.2988993

[22] Hu HW Yang JY Un CH Chen KY Huang CC Tsaih RH. The new method of feature selection for intradialytic hypotension prediction using machine learning. 2021 IEEE 3rd Eurasia Conference on Biomedical Engineering, Healthcare and Sustainability (ECBIOS)*,* Tainan, Taiwan, (2021). pp. 69–70. 10.1109/ECBIOS51820.2021.9510559

[23] BaeTW KimMS ParkJW KwonKK KimKH. Multi-layer perceptron-based real-time intradialytic hypotension prediction using patient baseline information and heart-rate variation. Int J Environ Res Public Health. (2022) 19:10373. 10.3390/ijerph19161037336012006 PMC9408052

[24] Nie Y Nguyen NH Sinthong P Kalagnanam J. A time series is worth 64 words: long-term forecasting with transformers. arXiv:2211.14730 (2023). 10.48550/arXiv.2211.14730

[25] Wu H Hu T Liu Y Zhou H Wang J Long M. TimesNet: temporal 2D-variation modeling for general time series analysis. arXiv:2210.02186 (2023). 10.48550/arXiv.2210.02186

[26] Bai S Kolter JZ Koltun V. An Empirical Evaluation of Generic Convolutional and Recurrent Networks for Sequence Modeling. arXiv:1803.01271 (2018). 10.48550/arXiv.1803.01271

[27] Trockman A Kolter JZ. Patches are all you need? arXiv:2201.09792 (2022). 10.48550/arXiv.2201.09792

[28] Janssens ACJW Martens FK. Reflection on modern methods: revisiting the area under the ROC curve. Int J Epidemiol. (2020) 49:1397–403. 10.1093/ije/dyz274 31967640

[29] Sundararajan M Taly A Yan Q. Axiomatic attribution for deep networks. arXiv:1703.01365 (2017). 10.48550/arXiv.1703.01365

[30] KeaneDF RaimannJG ZhangH WillettsJ ThijssenS KotankoP. The time of onset of intradialytic hypotension during a hemodialysis session associates with clinical parameters and mortality. Kidney Int. (2021) 99:1408–17. 10.1016/j.kint.2021.01.01833607178 PMC8165353

[31] SarsB van der SandeFM KoomanJP. Intradialytic hypotension: mechanisms and outcome. Blood Purif. (2020) 49:158–67. 10.1159/00050377631851975 PMC7114908

[32] KanbayM ErtugluLA AfsarB OzdoganE SiriopolD CovicA. An update review of intradialytic hypotension: concept, risk factors, clinical implications and management. Clin Kidney J. (2020) 13:981–93. 10.1093/ckj/sfaa07833391741 PMC7769545

[33] KeL ZhangH LongK PengZ HuangY MaX. Risk factors and prediction models for recurrent acute ischemic stroke: a retrospective analysis. PeerJ. (2024) 12:e18605. 10.7717/peerj.1860539611013 PMC11604039

[34] Loe A Murray S Wu Z. Random forest for dynamic risk prediction of recurrent events: a pseudo-observation approach. Biostatistics. (2025) 26:kxaf007. 10.1093/biostatistics/kxaf007 40083192

[35] AfentakisI UnsworthR HerreroP OliverN ReddyM GeorgiouP. Development and validation of binary classifiers to predict nocturnal hypoglycemia in adults with type 1 diabetes. J Diabetes Sci Technol. (2025) 19:153–60. 10.1177/1932296823118579637434362 PMC11696951

[36] Selby NM McIntyre CW. A systematic review of the clinical effects of reducing dialysate fluid temperature. Nephrol Dial Transplant. (2006) 21:1883–98. 10.1093/ndt/gfl126 16601075

[37] Daugirdas JT. Pathophysiology of dialysis hypotension: an update. Am J Kidney Dis. (2001) 38:S11–17. 10.1053/ajkd.2001.28090 11602456

