# Supplementary material for: Intra-Session recurrence in intradialytic hypotension prediction: evaluation implications and recurrence-aware modeling
Source: Front Digit Health. 2026 Jul 7;8:1804788. doi: 10.3389/fdgth.2026.1804788 (PMC13384919; doi:10.3389/fdgth.2026.1804788)
Supplement: Supplementary file 2 [file Datasheet2.pdf]

Supplementary Material:
"Improving Real-Time Prediction of Intradialytic Hypotension through Recurrence Pattern Integration and Evaluation Refinement"

Siun Kim[1,*], Jiwon Ryu[2,*], Sejoong Kim[2], Su Hwan Kim[3], Myeongju Kim[4] and Hyung-Jin Yoon[5,6]

[1]*Biomedical Research Institute, Seoul National University Hospital*

[2]*Department of Internal Medicine, Seoul National University College of Medicine*

[3]*Department of Information Statistics, Gyeongsang National University*

[4]*Center for Artificial Intelligence in Healthcare, Seoul National University Bundang Hospital*

[5]*Department of Human Systems Medicine, Seoul National University College of Medicine*

[6]*Medical Bigdata Research Center, Seoul National University College of Medicine*

[*]The authors are equally contributed.

[†]This research was supported by Ainex Corporation.

Table of content

1 Supplementary Methods

1-1 Data Preprocessing

Supplementary Table S1. Input covariates and their preprocessing method for intradialytic hypotension (IDH) prediction model development

1-2 Hyperparamter tuning

2 Supplementary Results

Supplementary Table S2. Baseline characteristics of study population (Full)

Supplementary Table S3. Predictive performance of baseline model on the definition of Nadir90

Supplementary Table S4. Predictive performance of baseline model on the definition of Nadir100

Supplementary Table S5. Predictive performance of baseline model on the definition of Fall20/MAP10

Supplementary Table S6. Predictive performance of baseline model on the definition of Fall20

Supplementary Table S7. Predictive performance of baseline model on the definition of Fall30

1 Supplementary Methods

1 – 1 Data Preprocessing

Our approach to feature selection and preprocessing for the intradialytic hypotension (IDH) prediction model was guided by clinical expertise, prior research, and careful consideration of potential confounding factors. This methodology aimed to mitigate issues such as shortcut learning and ensure the model's robustness and clinical relevance.

Categorical variables, including patient sex, diagnosis history, and prescription information, were binary encoded. Diagnosis history was categorized into all-time diagnoses, short-term diagnoses (within 180 days prior to the session), and diagnoses made before chronic kidney disease onset. Prescription information was classified into short-term (30 days) and long-term (180 days) usage patterns. To maintain model parsimony, features with less than 10% representation in either category (0 or 1) in the training dataset were excluded. Disease classifications were based on ICD-10 codes, evaluated by the date of first diagnosis relative to the session date. Drug classifications were determined by active ingredient names. All categorical variables were pre-screened by a clinical expert (author JR) based on their clinical relevance to IDH occurrence.

Continuous variables, particularly laboratory data, were processed using a Last Observation Carried Forward (LOCF) approach. We selected laboratory tests with an average LOCF period within 14 days from the Clinical Data Warehouse, further refined through clinical review for relevance to IDH pathophysiology. Based on previous research [1] highlighting the importance of prior session information, we incorporated baseline and vital sign data (SBP, DBP, heart rate) from the immediately preceding session.

Environmental factors, including day of the week [2], temperature [3], and air quality, were included based on literature supporting their influence on IDH occurrence. The day of the week was sine-transformed to reflect weekly cyclical patterns. Hourly temperature and PM2.5 levels for the hospital's location were obtained from the Korea Meteorological Administration's open MET data portal and aligned with session timing using LOCF.

Time-varying covariates were handled differently based on their characteristics. Vital signs (SBP, DBP, heart rate) along with their measurement times were input as time-series data into a Long Short-Term Memory (LSTM) model to capture temporal dynamics and address irregular sampling. The time to predict variable, however, was processed through a Multi-Layer Perceptron (MLP) due to its distinct nature. All data underwent robust scaling to enhance the model's resilience to outliers, using sklearn's RobustScaler on the training dataset.

1 – 2 Hyperparamter tuning

For the LSTM baseline, the learning rate was set between 1e-3 and 1e-4, with weight decay of 1e-3 or none. We used CrossEntropyLoss with the Adam optimizer, trained for 100 epochs, applying MultiStepLR (halved at epochs 50 and 66) and early stopping (patience of 15). The architecture used 256 hidden units for both

time-invariant and time-varying features with 8 LSTM layers as default.

For LSTMwithAttention, TCN, and ConvMixer, the same training strategy was applied. Learning rates were set to 1e-4, 1e-3, and 5e-4, respectively. LSTMwithAttention followed the same default dimensions as LSTM, while TCN and ConvMixer used default settings without additional tuning.

Supplementary Table S1. Input covariates and their preprocessing method for intradialytic hypotension (IDH) prediction model development

Main category	Subcategory	Input covariates	Preprocessing methods
**Patient Characteristics**	**Demographics**	Sex	Binary encoding (0: female, 1: male)
		Age	Calculated from birth date and session date
		BMI	Calculated from height and pre-dialysis weight
	**Medical History**	Diagnosis history	Binary encoding for presence/absence, categorized as all-time, short-term (180 days), and pre-CKD onset<br>- Used diagnosis categories: AKI, CVD, CANCER, COMP, DM, GMNR, LUNGD, PSYCD, STROKE
		Prescription information	Binary encoding for presence/absence, categorized as short-term (30 days) and long-term (180 days)<br>- antihypertensive, anticoagulants, diuretics, alpha-blocker, beta-blocker, ARBs, CCB, EPO, DOAC, VKA, insulin, sulfonylureas, DPP4i, heparin, antiosteoporosis, PPI, NSAIDs, hyperkalemia, phosphate-binder, antiparathyroid, antianemia, constipation, antigout, antibiotic
	**Laboratory Results**	Hematology and Chemistry	Hct, PLT, Hb, eGFR, Creatinine, BUN, Alkaline phosphatase, Phosphorus, Bilirubin, Uric Acid, Cholesterol, Calcium, CRP, Potassium, GPT, GOT, Protein, Albumin, Chloride, Sodium, CO_2; LOCF from measurements prior to dialysis session
**Dialysis Information**	**Pre-dialysis Measurements**	Pre-dialysis weight	Extracted from TDMS NTS data
		Target weight	Extracted from TDMS NTS data
		Interdialytic weight change	Calculated from current and previous session weights
		Days since last dialysis	Calculated from current and previous session dates
	**Baseline Vitals**	Baseline SBP, DBP, heart rate	Extracted baseline value from first two non-NaN values within 15 minutes, or first available if not
	**Previous Session Data**	Mean/Baseline SBP, DBP, heart rate from previous session	Calculated from previous session's TDMS TS data +
		Previous session IDH occurrences	Binary encoding for each IDH type from previous session's data
	**Dialysis Prescription**	Heparin dosage, UF volume, UF rate, UF total time, Dialysate flow, Dialysate temperature, Dialysate sodium concentration	Extracted from TDMS TS data, LOCF for missing values
	**Dialyzer Information**	Dialyzer type, flux type, surface area, UF coefficient	Encoded based on dialyzer characteristics
	**HD Scores**	spKt/V, eKt/V, nPCR, URR,	Extracted from patient scores data

		residual kidney function,	
**Temporal Factors**	**Session Timing**	Week day<br>AM/PM indicator	Applied sine transformation of weekday (0-6)<br>Binary encoding (0: AM, 1: PM)
**Environmental Factors**	**Weather**	Temperature	LOCF from hourly data
	**Air Quality**	PM2.5	LOCF from hourly data
**Time-varying Data**	**Time to Predict**	Time to predict	Comprised the time elapsed from dialysis start to the prediction point, measured in seconds
	**Vital Signs**	Time to measure, SBP, DBP, Heart rate	Extracted from TDMS TS data

IDH, Intradialytic Hypotension; BMI, Body Mass Index; CKD, Chronic Kidney Disease; AKI, Acute Kidney Injury; CVD, Cardiovascular Disease; COMP, Cardiovascular Device Complications; DM, Diabetes Mellitus; GMNR, Glomerulonephritis; LUNGD, Lung Disease; PSYCD, Psychiatric Disease; STROKE, Stroke; ARBs, Angiotensin Receptor Blockers; CCB, Calcium Channel Blockers; EPO, Erythropoietin; DOAC, Direct Oral Anticoagulants; VKA, Vitamin K Antagonists; DPP4i, Dipeptidyl Peptidase-4 inhibitors; PPI, Proton Pump Inhibitors; NSAIDs, Nonsteroidal Anti-inflammatory Drugs; Hct, Hematocrit; PLT, Platelets; Hb, Hemoglobin; eGFR, estimated Glomerular Filtration Rate; BUN, Blood Urea Nitrogen; CRP, C-Reactive Protein; GPT, Glutamic Pyruvic Transaminase; GOT, Glutamic Oxaloacetic Transaminase; SBP, Systolic Blood Pressure; DBP, Diastolic Blood Pressure; UF, Ultrafiltration; Kt/V, Dialysis Adequacy Measure; spKt/V, single-pool Kt/V; eKt/V, equilibrated Kt/V; URR, Urea Reduction Ratio; nPCR, normalized Protein Catabolic Rate; PM2.5, Particulate Matter 2.5; TDMS, Therapy Data Management System; NTS, Non-Time Series; TS, Time Series; LOCF, Last Observation Carried Forward

2 Supplementary Results

Supplementary Table S2 presents the detailed baseline characteristics of the study population, including both the training and test sets. Tables S3-S7 report the predictive performance of various model types across different IDH definitions, providing comprehensive metrics such as accuracy, precision, recall, F1 score, AUROC, AUPRC, MCC, and NPV.

Supplementary Table S2. Baseline characteristics of study population (Full)

	Train (*N*=52)	Test (*N*=14)	Total (N=66)
**Total sessions, n**	10,179	2,588	12,767
**Session number per patient, n**	195.8 (78.5)	185.9 (94.4)	193.4 (82.2)
**Age, yr†**	67.1 (15.7)	70.2 (10.8)	68.2 (14.4)
**Sex, n (%)**			
Female	23 (44.2)	6 (42.9)	29 (43.9)
Male	29 (55.8)	8 (57.1)	37 (56.1)
**BMI, kg/m^2†**	22.5 (2.7)	23.5 (3.2)	22.7 (2.9)
**IDH occurrence, %†**			
Nadir90	9.8 (14.5)	8.1 (10.9)	9.4 (13.8)
Nadir100	17.6 (19.7)	16.7 (24.2)	17.4 (20.7)
Fall20/MAP10	67.2 (24.2)	68.4 (22.4)	67.5 (23.9)
Fall20	58.3 (26.7)	61.0 (26.0)	58.9 (26.6)
Fall30	40.1 (25.6)	43.3 (28.2)	40.8 (26.2)
**Baseline vital measurements†**			
Systolic BP, mm Hg	144.7 (17.5)	149.9 (12.9)	145.8 (16.8)
Diastolic BP, mm Hg	65.5 (11.7)	68.7 (10.4)	66.2 (11.5)
Heart rate, bpm	72.5 (9.4)	71.2 (10.3)	72.2 (9.7)
**Dialysis cause, %†**			
Diabetes Mellitus	23.1 (42.1)	14.3 (35.0)	21.2 (40.9)

Hypertension	15.4 (36.1)	28.6 (45.2)	18.2 (38.6)
Immune-related	13.5 (34.1)	7.1 (25.8)	12.1 (32.6)
Other	48.1 (50.0)	50.0 (50.0)	48.5 (50.0)
**Dialysis settings†**			
Weight			
Pre-dialytic weight, kg	59.6 (9.8)	62.2 (12.8)	60.2 (10.6)
Dry wieght, kg	57.8 (9.6)	60.1 (12.7)	58.3 (10.4)
Target weight, kg	57.9 (9.7)	60.1 (12.7)	58.4 (10.4)
Interdialytic weight change, kg	1.7 (1.1)	2.1 (0.6)	1.8 (1.0)
UF settings			
UF volume, mL	1903.1 (770.5)	2253.1 (616.0)	1977.3 (754.2)
UF total time, seconds	237.1 (9.7)	238.8 (2.7)	237.5 (8.7)
UF rate, mL/kg/hour	460.7 (176.4)	520.5 (135.2)	473.4 (170.2)
Dialysis mode, %			
HDF	65.8 (44.7)	71.4 (54.8)	67.0 (44.8)
HD or HDx	34.2 (44.7)	28.6 (45.2)	33.0 (44.8)
Dialysis adequacy			
spKt/V, (unitless)	1.8 (0.3)	1.5 (0.2)	1.8 (0.3)
eKt/V, (unitless)	1.6 (0.2)	0.8 (0.2)	1.6 (0.2)
nPCR, g/kg/day	0.8 (0.1)	76.2 (5.4)	0.8 (0.1)
URR, %	77.5 (4.6)	60.1 (12.7)	77.2 (4.8)
Residual Renal Function, %	7.0 (12.0)	13.2 (26.7)	8.3 (16.5)
Flux type, %			
High	97.5 (13.0)	100.0 (0.0)	98.1 (11.6)
Low	2.5 (13.0)	0.0 (0.0)	1.9 (11.6)
Dialyzer			
Surface area, m^2	1.6 (0.2)	1.7 (0.2)	1.6 (0.2)
UF coeffient, mL/hour/mm Hg	52.9 (14.3)	59.4 (14.3)	54.3 (14.5)
BFV, mL	90.9 (15.7)	96.4 (17.3)	92.0 (16.2)
Dialysis vintage, month	90.8 (57.5)	71.7 (41.6)	86.8 (55.1)
Vein Type, %			
AVF	85.6 (33.1)	71.4 (45.2)	82.6 (36.4)
AVG	3.8 (19.2)	14.3 (35.0)	6.0 (23.8)
Session Timing, %			
A.M.	53.3 (46.8)	64.3 (47.5)	55.6 (47.2)
P.M.	46.7 (46.8)	35.7 (47.5)	44.4 (47.2)
**Diagnosis, %†**			
AKI	13.5 (34.1)	21.0 (41.0)	15.2 (35.9)
Cancer	19.6 (39.3)	42.0 (49.5)	24.6 (42.8)
Complications associated with medical devices	47.0 (49.1)	50.0 (50.0)	47.6 (49.3)
Diabetes Mellitus	42.9 (49.1)	35.7 (47.9)	41.4 (48.9)
Glomerulonephritis	28.8 (45.3)	26.8 (42.7)	28.4 (44.8)
Hypertension	43.5 (48.3)	35.7 (47.9)	41.9 (48.3)
Lung Disease	40.3 (49.0)	42.9 (49.5)	40.8 (49.1)
Stroke	31.7 (45.8)	21.4 (41.0)	29.5 (45.0)
**Prescription within 30 days, %†**			
Anti-hypertensive			
Alpha Blockers	53.7 (42.1)	40.5 (42.6)	50.9 (42.6)

Beta Blockers	42.1 (44.3)	22.9 (37.6)	38.0 (43.7)
ARBs	62.4 (39.9)	55.2 (45.0)	60.9 (41.1)
CCBs	75.6 (33.9)	71.3 (37.0)	74.7 (34.6)
Diuretics	37.3 (43.9)	44.8 (44.1)	38.9 (44.1)
Anticoagulants			
DOACs	7.6 (26.2)	7.0 (25.1)	7.4 (25.9)
VKAs	3.8 (19.2)	0.0 (0.0)	3.0 (17.1)
Heparin	8.2 (15.9)	12.9 (29.9)	9.2 (19.8)
Erythropoietin	96.2 (7.5)	96.6 (4.9)	96.3 (7.1)
Diabetes Medications			
Insulin	12.4 (28.4)	14.9 (33.6)	12.9 (29.6)
Sulfonylureas	15.9 (34.0)	14.2 (34.8)	15.6 (34.2)
DPP-4i	26.9 (42.7)	21.3 (40.7)	25.7 (42.4)
Anti-Osteoporosis	39.5 (40.5)	45.1 (39.4)	40.7 (40.3)
Phosphate Binders	84.4 (30.2)	92.2 (16.5)	86.0 (28.0)
Anti-Parathyroid Medications	46.0 (45.5)	56.1 (48.6)	48.2 (46.3)
Hyperkalemia Treatments	67.0 (42.5)	66.8 (36.2)	66.9 (41.3)
Anti-Anemia Medications	19.3 (38.2)	21.3 (40.5)	19.7 (38.7)
PPIs	51.4 (44.0)	56.3 (42.2)	52.5 (43.7)
Constipation Treatments	44.5 (40.8)	65.7 (36.1)	49.0 (40.8)
NSAIDs	13.3 (23.8)	11.5 (25.6)	12.9 (24.2)
Anti-Gout Medications	27.4 (43.3)	49.9 (49.5)	32.2 (45.6)
Antibiotics	14.3 (24.3)	14.2 (23.6)	14.3 (24.2)
**Laboratory test results†**			
Hct, %	33.2 (2.4)	32.2 (2.9)	33.0 (2.6)
Hb, g/dl	10.6 (0.8)	10.4 (1.0)	10.6 (0.8)
PLT, 10^3/uL	164.2 (44.7)	162.0 (61.5)	163.7 (48.8)
eGFR, mL/min/1.73m^2	7.0 (3.3)	6.3 (1.2)	6.9 (3.0)
Creatinine, mg/dL	8.1 (2.4)	8.0 (1.6)	8.0 (2.2)
BUN, mg/dL	17.3 (7.6)	21.6 (18.4)	18.2 (11.0)
Sodium, mEq/L	137.8 (2.2)	137.2 (2.2)	137.7 (2.2)
Potassium, mmol/L	4.5 (0.5)	4.6 (0.4)	4.5 (0.5)
Calcium, mg/dL	9.0 (0.5)	9.2 (0.6)	9.1 (0.6)
Phosphorus, mg/dL	4.1 (0.8)	4.3 (0.7)	4.1 (0.8)
Chloride, mEq/L	98.6 (2.9)	97.7 (3.3)	98.4 (3.0)
CO2, mEq/L	25.6 (2.1)	25.9 (2.4)	25.7 (2.2)
GPT (ALT), IU/L	16.5 (10.1)	15.3 (6.4)	16.2 (9.4)
GOT (AST), IU/L	20.1 (8.5)	19.3 (5.8)	20.0 (8.0)
Alkaline phosphatase, IU/L	102.5 (56.5)	94.5 (30.8)	100.8 (52.2)
Protein, g/dl	6.8 (0.5)	6.5 (0.5)	6.7 (0.5)
Albumin, g/dl	3.8 (0.4)	3.8 (0.3)	3.8 (0.3)
hsCRP, mg/dL	0.6 (0.8)	0.4 (0.3)	0.5 (0.7)
Uric Acid, mg/dL	5.7 (1.1)	5.1 (1.2)	5.6 (1.1)
Cholesterol, mg/dL	136.2 (26.2)	148.0 (35.3)	138.7 (28.8)

SD, Standard Deviation; BMI, Body Mass Index; IDH, Intra-dialytic Hypotension; Nadir90, nadir systolic blood pressure (SBP) <90 mm Hg; Nadir100, nadir SBP <100 mm Hg; Fall20/MAP10, a decrease in SBP by ≥20 mm Hg or a decrease in mean arterial pressure >10 mm Hg from baseline; Fall20, a decrease in SBP ≥20 mm Hg from baseline; Fall30, a decrease in SBP ≥30 mm Hg from baseline; BP, Blood Pressure; bpm, beats per minute; HDF, Hemodiafiltration; HD, Hemodialysis; HDx, Expanded Hemodialysis; UF, Ultrafiltration; spKt/V, single-pooled Kt/V; eKt/V, Equilibrated Kt/V; nPCR, Normalized Protein Catabolic Rate; URR, Urea Reduction Ratio; AVF, Arteriovenous Fistula; AVG, Arteriovenous Graft; Hct, Hematocrit; Hb,

Hemoglobin; PLT, Platelet; eGFR, estimated Glomerular Filtration Rate; BUN, Blood Urea Nitrogen; Sodium, GPT (ALT), Glutamate Pyruvate Transaminase (Alanine Aminotransferase); GOT (AST), Glutamate Oxaloacetate Transaminase (Aspartate Aminotransferase); hsCRP, high-sensitivity C-reactive Protein.
†To account for the variation in the number of dialysis sessions per patient, we calculated the proportions within each session and then reported the mean and standard deviation of these proportions at the patient level.

Supplementary Table S3. Predictive performance of baseline model on the definition of Nadir90

Model types	Performance metrics							
**IDH occurrence type**	**Accuracy**	**Precision**	**Recall**	**F1**	**AUROC**	**AUPRC**	**MCC**	**NPV**
Naïve baseline								
All	0.977 [0.976, 0.980]	0.034 [0.012, 0.065]	0.032 [0.010, 0.062]	0.033 [0.011, 0.064]	0.510 [0.500, 0.526]	0.013 [0.011, 0.015]	0.022 [-0.001, 0.053]	0.988 [0.987, 0.990]
Initial	**0.994 [0.992, 0.995]**	0.000 [0.000, 0.000]	0.000 [0.000, 0.000]	0.000 [0.000, 0.000]	0.500 [0.500, 0.500]	0.006 [0.005, 0.008]	0.000 [0.000, 0.000]	0.994 [0.992, 0.995]
Recurrent	0.630 [0.595, 0.662]	0.034 [0.012, 0.065]	0.125 [0.041, 0.204]	0.054 [0.018, 0.101]	0.599 [0.549, 0.642]	0.078 [0.061, 0.096]	-0.120 [-0.173, -0.065]	0.894 [0.870, 0.918]
Baseline								
All	0.961 [0.958, 0.965]	0.145 [0.118, 0.172]	**0.441 [0.372, 0.508]**	**0.218 [0.181, 0.256]**	**0.936 [0.921, 0.948]**	**0.156 [0.115, 0.198]**	**0.238 [0.195, 0.278]**	**0.993 [0.992, 0.994]**
Initial	0.975 [0.972, 0.977]	0.111 [0.078, 0.147]	**0.333 [0.241, 0.424]**	0.166 [0.119, 0.214]	**0.929 [0.907, 0.947]**	**0.102 [0.068, 0.144]**	**0.182 [0.128, 0.232]**	**0.995 [0.994, 0.996]**
Recurrent	0.703 [0.664, 0.742]	0.119 [0.068, 0.171]	0.475 [0.306, 0.629]	0.191 [0.113, 0.263]	0.749 [0.693, 0.801]	0.188 [0.100, 0.306]	0.113 [0.012, 0.201]	0.945 [0.921, 0.966]
Single-task								
All	NA	NA	NA	NA	NA	NA	NA	NA
Initial	0.983 [0.981, 0.985]	0.108 [0.064, 0.158]	0.161 [0.097, 0.232]	0.129 [0.078, 0.184]	0.809 [0.761, 0.853]	0.053 [0.036, 0.079]	0.123 [0.072, 0.179]	0.994 [0.992, 0.995]
Recurrent	0.750 [0.714, 0.787]	0.187 [0.126, 0.253]	0.718 [0.581, 0.850]	0.296 [0.212, 0.382]	0.775 [0.708, 0.831]	0.163 [0.108, 0.242]	0.272 [0.184, 0.360]	0.971 [0.953, 0.987]
With IDH occurrence info. (binary)								
All	**0.984 [0.982, 0.986]**	**0.248 [0.169, 0.321]**	0.165 [0.110, 0.216]	0.198 [0.135, 0.252]	0.845 [0.817, 0.869]	0.101 [0.072, 0.137]	0.194 [0.131, 0.249]	0.990 [0.988, 0.991]
Initial	0.989 [0.987, 0.990]	**0.176 [0.096, 0.258]**	0.135 [0.075, 0.200]	0.153 [0.084, 0.220]	0.818 [0.781, 0.857]	0.061 [0.039, 0.094]	0.149 [0.079, 0.216]	0.993 [0.992, 0.995]
Recurrent	0.917 [0.895, 0.941]	0.333 [0.100, 0.589]	0.125 [0.030, 0.238]	0.182 [0.047, 0.321]	0.728 [0.656, 0.796]	0.194 [0.106, 0.316]	0.167 [0.021, 0.325]	0.934 [0.913, 0.955]
With detailed IDH occurrence information								
All	0.975 [0.972, 0.977]	0.160 [0.120, 0.207]	0.250 [0.191, 0.314]	0.195 [0.150, 0.246]	0.790 [0.756, 0.823]	0.071 [0.053, 0.095]	0.188 [0.141, 0.239]	0.991 [0.989, 0.992]
Initial	0.981 [0.979, 0.983]	0.066 [0.034, 0.102]	0.117 [0.059, 0.176]	0.085 [0.044, 0.127]	0.722 [0.669, 0.768]	0.026 [0.017, 0.042]	0.079 [0.036, 0.123]	0.993 [0.992, 0.994]
Recurrent	0.875 [0.845, 0.900]	0.306 [0.198, 0.413]	0.550 [0.391, 0.703]	0.393 [0.269, 0.500]	0.875 [0.830, 0.916]	0.399 [0.256, 0.567]	0.347 [0.222, 0.464]	0.962 [0.943, 0.979]
Loss weighting								
All	NA	NA	NA	NA	NA	NA	NA	NA
Initial	0.987 [0.985, 0.989]	0.167 [0.105, 0.235]	0.180 [0.114, 0.250]	**0.173 [0.109, 0.240]**	0.797 [0.747, 0.849]	0.079 [0.052, 0.119]	0.167 [0.103, 0.234]	0.994 [0.993, 0.995]
Recurrent	0.869 [0.841, 0.897]	0.262 [0.164, 0.369]	0.425 [0.268, 0.581]	0.324 [0.214, 0.438]	0.759 [0.685, 0.833]	0.214 [0.128, 0.337]	0.265 [0.148, 0.394]	0.952 [0.933, 0.970]
Loss weighting with detailed IDH occurrence info.								
All	NA	NA	NA	NA	NA	NA	NA	NA
Initial	0.992 [0.991, 0.993]	0.000 [0.000, 0.000]	0.000 [0.000, 0.000]	0.000 [0.000, 0.000]	0.582 [0.529, 0.628]	0.009 [0.007, 0.011]	-0.002 [-0.002, -0.001]	0.992 [0.991, 0.994]
Recurrent	**0.921 [0.899, 0.943]**	**0.462 [0.297, 0.622]**	0.450 [0.293, 0.611]	**0.456 [0.306, 0.582]**	**0.914 [0.882, 0.942]**	**0.448 [0.287, 0.591]**	0.413 [0.262, 0.549]	0.956 [0.938, 0.974]

Nadir90, nadir systolic blood pressure (SBP) <90 mm Hg; IDH: Intradialytic Hypotension; AUROC: Area Under the Receiver Operating Characteristic curve; AUPRC: Area Under the Precision-Recall Curve; MCC: Matthews Correlation Coefficient; NPV: Negative Predictive Value; NA: Not Applicable; info.: information.

† Bold values indicate the highest performance, while underlined values show the second-best performance for each metric.

Supplementary Table S4. Predictive performance of baseline model on the definition of Nadir100

Model types	Performance metrics							
**IDH occurrence type**	**Accuracy**	**Precision**	**Recall**	**F1**	**AUROC**	**AUPRC**	**MCC**	**NPV**
Naïve baseline								
All	0.950 [0.946, 0.954]	0.123 [0.097, 0.164]	0.058 [0.045, 0.074]	0.079 [0.061, 0.099]	0.521 [0.514, 0.529]	0.042 [0.038, 0.049]	0.061 [0.041, 0.084]	0.965 [0.961, 0.969]
Initial	**0.986 [0.984, 0.988]**	0.000 [0.000, 0.000]	0.000 [0.000, 0.000]	0.000 [0.000, 0.000]	0.500 [0.500, 0.500]	0.014 [0.012, 0.016]	0.000 [0.000, 0.000]	0.986 [0.984, 0.988]
Recurrent	0.568 [0.544, 0.600]	0.123 [0.097, 0.164]	0.114 [0.090, 0.141]	0.118 [0.092, 0.150]	0.572 [0.556 ,0.599]	0.239 [0.215, 0.262]	-0.167 [-0.203, -0.120]	0.706 [0.681, 0.744]
Baseline								
All	**0.970 [0.967, 0.972]**	**0.586 [0.546, 0.626]**	0.611 [0.569, 0.651]	**0.598 [0.565, 0.633]**	**0.976 [0.973, 0.980]**	**0.631 [0.591, 0.670]**	**0.582 [0.548, 0.618]**	0.985 [0.983, 0.987]
Initial	0.984 [0.982, 0.986]	**0.537 [0.465, 0.607]**	0.400 [0.341, 0.458]	**0.459 [0.400, 0.515]**	**0.977 [0.972, 0.982]**	**0.462 [0.399, 0.526]**	**0.456 [0.398, 0.513]**	0.990 [0.988, 0.991]
Recurrent	**0.820 [0.797, 0.843]**	0.607 [0.559, 0.661]	0.777 [0.726, 0.824]	**0.682 [0.641, 0.722]**	**0.899 [0.880, 0.918]**	**0.746 [0.690, 0.795]**	**0.567 [0.514, 0.620]**	0.919 [0.898, 0.939]
Single-task								
All	NA	NA	NA	NA	NA	NA	NA	NA
Initial	0.973 [0.971, 0.976]	0.311 [0.266, 0.357]	0.489 [0.429, 0.550]	0.380 [0.331, 0.427]	0.955 [0.942, 0.966]	0.341 [0.275, 0.399]	0.377 [0.327, 0.425]	0.991 [0.990, 0.993]
Recurrent	0.764 [0.739, 0.790]	0.518 [0.463, 0.570]	0.720 [0.660, 0.773]	0.603 [0.552, 0.644]	0.839 [0.812, 0.864]	0.632 [0.571, 0.689]	0.453 [0.392, 0.509]	0.894 [0.871, 0.916]
With IDH occurrence info. (binary)								
All	0.961 [0.958, 0.964]	0.480 [0.445, 0.514]	**0.694 [0.654, 0.730]**	0.568 [0.536, 0.597]	0.971 [0.966, 0.975]	0.587 [0.545, 0.631]	0.558 [0.527, 0.589]	**0.988 [0.986, 0.990]**
Initial	0.978 [0.975, 0.980]	0.379 [0.326, 0.435]	**0.528 [0.467, 0.592]**	0.441 [0.388, 0.494]	0.969 [0.961, 0.976]	0.390 [0.327, 0.464]	0.436 [0.386, 0.491]	**0.992 [0.990, 0.993]**
Recurrent	0.792 [0.769, 0.816]	0.555 [0.506, 0.602]	0.810 [0.761, 0.856]	0.659 [0.615, 0.697]	0.884 [0.863, 0.903]	0.722 [0.665, 0.771]	0.536 [0.483, 0.586]	0.926 [0.906, 0.945]
With detailed IDH occurrence information								
All	0.960 [0.956, 0.962]	0.465 [0.429, 0.496]	0.680 [0.639, 0.717]	0.553 [0.520, 0.581]	0.961 [0.954, 0.967]	0.526 [0.479, 0.573]	0.543 [0.509, 0.572]	0.988 [0.986, 0.989]
Initial	0.982 [0.980, 0.985]	0.457 [0.385, 0.528]	0.340 [0.280, 0.400]	0.390 [0.332, 0.449]	0.947 [0.933, 0.959]	0.323 [0.266, 0.390]	0.386 [0.327, 0.445]	0.989 [0.987, 0.991]
Recurrent	0.710 [0.682, 0.737]	0.459 [0.420, 0.499]	**0.967 [0.944, 0.988]**	0.623 [0.585, 0.660]	0.883 [0.862, 0.901]	0.669 [0.610, 0.728]	0.512 [0.474, 0.550]	**0.983 [0.972, 0.994]**
Loss weighting								
All	NA	NA	NA	NA	NA	NA	NA	NA
Initial	0.977 [0.975, 0.980]	0.325 [0.267, 0.384]	0.336 [0.277, 0.396]	0.331 [0.274, 0.384]	0.941 [0.927, 0.954]	0.278 [0.225, 0.336]	0.319 [0.264, 0.373]	0.989 [0.987, 0.990]
Recurrent	0.820 [0.798, 0.841]	**0.609 [0.561, 0.659]**	0.769 [0.716, 0.817]	0.680 [0.636, 0.722]	0.883 [0.861, 0.903]	0.711 [0.654, 0.762]	0.564 [0.511, 0.616]	0.917 [0.896, 0.935]
Loss weighting with detailed IDH occurrence info.								
All	NA	NA	NA	NA	NA	NA	NA	NA
Initial	0.977 [0.974, 0.979]	0.309 [0.253, 0.367]	0.328 [0.272, 0.389]	0.318 [0.265, 0.369]	0.937 [0.920, 0.952]	0.269 [0.218, 0.331]	0.306 [0.253, 0.358]	0.989 [0.987, 0.990]
Recurrent	0.804 [0.782, 0.828]	0.574 [0.523, 0.623]	0.813 [0.764, 0.858]	0.673 [0.629, 0.715]	0.893 [0.872, 0.912]	0.705 [0.640, 0.767]	0.555 [0.502, 0.608]	0.929 [0.910, 0.948]

Nadir100, nadir SBP <100 mm Hg; IDH: Intradialytic Hypotension; AUROC: Area Under the Receiver Operating Characteristic curve; AUPRC: Area Under the Precision-Recall Curve; MCC: Matthews Correlation Coefficient; NPV: Negative Predictive Value; NA: Not Applicable; info.: information.

† Bold values indicate the highest performance, while underlined values show the second-best performance for each metric.

Supplementary Table S5. Predictive performance of baseline model on the definition of Fall20/MAP10

Model types	Performance metrics							
IDH occurrence type	Accuracy	Precision	Recall	F1	AUROC	AUPRC	MCC	NPV
Naïve baseline								
All	0.817 [0.812, 0.823]	0.784 [0.774, 0.797]	0.730 [0.718, 0.741]	0.756 [0.748, 0.766]	0.801 [0.796, 0.808]	0.677 [0.666, 0.688]	0.611 [0.601, 0.623]	0.836 [0.830, 0.843]
Initial	0.909 [0.905, 0.918]	0.000 [0.000, 0.000]	0.000 [0.000, 0.000]	0.000 [0.000, 0.000]	0.500 [0.500, 0.500]	0.091 [0.082, 0.095]	0.000 [0.000, 0.000]	0.909 [0.905, 0.918]
Recurrent	0.742 [0.734, 0.751]	0.784 [0.774, 0.797]	0.836 [0.827, 0.847]	0.809 [0.802, 0.817]	0.700 [0.690, 0.709]	0.763 [0.753, 0.775]	0.414 [0.395, 0.432]	0.644 [0.630, 0.663]
Baseline								
All	0.862 [0.856, 0.868]	0.802 [0.792, 0.812]	0.856 [0.847, 0.865]	0.828 [0.821, 0.835]	0.938 [0.934, 0.942]	0.915 [0.909, 0.920]	0.714 [0.702, 0.725]	0.905 [0.898, 0.911]
Initial	0.909 [0.902, 0.915]	0.554 [0.514, 0.594]	0.461 [0.422, 0.497]	0.503 [0.468, 0.534]	0.878 [0.864, 0.891]	0.537 [0.500, 0.572]	0.456 [0.418, 0.490]	0.941 [0.935, 0.947]
Recurrent	0.820 [0.812, 0.829]	0.828 [0.818, 0.838]	0.914 [0.907, 0.921]	0.869 [0.863, 0.876]	0.898 [0.892, 0.905]	0.944 [0.940, 0.949]	0.590 [0.572, 0.608]	0.798 [0.782, 0.813]
Single-task								
All	NA	NA	NA	NA	NA	NA	NA	NA
Initial	0.879 [0.871, 0.886]	0.426 [0.396, 0.459]	**0.582 [0.548, 0.618]**	0.492 [0.464, 0.522]	0.859 [0.845, 0.872]	0.504 [0.467, 0.540]	0.432 [0.402, 0.463]	**0.951 [0.946, 0.956]**
Recurrent	0.805 [0.795, 0.814]	0.806 [0.795, 0.816]	0.930 [0.923, 0.938]	0.864 [0.856, 0.871]	0.879 [0.871, 0.887]	0.935 [0.929, 0.940]	0.543 [0.522, 0.563]	0.800 [0.780, 0.819]
With IDH occurrence info. (binary)								
All	0.861 [0.855, 0.866]	0.790 [0.780, 0.800]	**0.873 [0.864, 0.881]**	0.829 [0.822, 0.837]	0.938 [0.935, 0.942]	0.915 [0.909, 0.920]	0.714 [0.703, 0.725]	**0.913 [0.907, 0.919]**
Initial	0.911 [0.905, 0.917]	0.557 [0.524, 0.593]	0.545 [0.513, 0.581]	0.551 [0.524, 0.582]	0.887 [0.876, 0.898]	0.554 [0.518, 0.591]	0.501 [0.472, 0.533]	0.949 [0.944, 0.954]
Recurrent	0.816 [0.807, 0.824]	0.820 [0.810, 0.829]	0.921 [0.914, 0.928]	0.867 [0.860, 0.874]	0.896 [0.889, 0.903]	0.943 [0.939, 0.948]	0.579 [0.560, 0.597]	0.804 [0.787, 0.821]
With detailed IDH occurrence information								
All	**0.872 [0.867, 0.878]**	**0.825 [0.815, 0.834]**	0.853 [0.843, 0.861]	0.839 [0.831, 0.846]	**0.945 [0.941, 0.948]**	**0.921 [0.916, 0.926]**	**0.734 [0.722, 0.744]**	0.905 [0.898, 0.910]
Initial	**0.916 [0.909, 0.923]**	**0.640 [0.596, 0.683]**	0.385 [0.350, 0.420]	**0.481 [0.446, 0.516]**	0.893 [0.880, 0.904]	0.556 [0.521, 0.593]	0.455 [0.419, 0.489]	0.934 [0.928, 0.940]
Recurrent	0.833 [0.825, 0.841]	**0.839 [0.830, 0.849]**	0.921 [0.915, 0.929]	0.878 [0.872, 0.885]	0.908 [0.902, 0.914]	0.949 [0.944, 0.953]	0.620 [0.603, 0.639]	0.817 [0.801, 0.832]
Loss weighting								
All	NA	NA	NA	NA	NA	NA	NA	NA
Initial	0.907 [0.901, 0.914]	0.543 [0.507, 0.583]	0.500 [0.461, 0.537]	0.521 [0.488, 0.554]	0.872 [0.858, 0.886]	0.543 [0.507, 0.580]	0.470 [0.435, 0.506]	0.945 [0.939, 0.950]
Recurrent	0.822 [0.814, 0.830]	0.833 [0.823, 0.843]	0.911 [0.904, 0.919]	0.870 [0.864, 0.877]	0.898 [0.891, 0.904]	0.944 [0.939, 0.949]	0.596 [0.578, 0.614]	0.795 [0.781, 0.812]
Loss weighting with detailed IDH occurrence info.								
All	NA	NA	NA	NA	NA	NA	NA	NA
Initial	**0.916 [0.909, 0.923]**	0.596 [0.562, 0.633]	0.515 [0.480, 0.550]	**0.553 [0.522, 0.582]**	**0.901 [0.889, 0.912]**	**0.578 [0.540, 0.612]**	**0.508 [0.476, 0.539]**	0.947 [0.941, 0.952]
Recurrent	**0.837 [0.829, 0.845]**	0.836 [0.827, 0.845]	**0.935 [0.928, 0.942]**	**0.883 [0.876, 0.889]**	**0.921 [0.915, 0.927]**	**0.957 [0.953, 0.961]**	**0.631 [0.613, 0.648]**	**0.841 [0.826, 0.855]**

Fall20/MAP10, a decrease in SBP by ≥20 mm Hg or a decrease in mean arterial pressure >10 mm Hg from baseline; IDH: Intradialytic Hypotension; AUROC: Area Under the Receiver Operating Characteristic curve; AUPRC: Area Under the Precision-Recall Curve; MCC: Matthews Correlation Coefficient; NPV: Negative Predictive Value; NA: Not Applicable; info.: information.

† Bold values indicate the highest performance, while underlined values show the second-best performance for each metric.

Supplementary Table S6. Predictive performance of baseline model on the definition of Fall20

Model types	Performance metrics							
**IDH occurrence type**	**Accuracy**	**Precision**	**Recall**	**F1**	**AUROC**	**AUPRC**	**MCC**	**NPV**
Naïve baseline								
All	0.829 [0.823, 0.835]	0.770 [0.760, 0.781]	0.715 [0.703, 0.727]	0.742 [0.732, 0.751]	0.802 [0.794, 0.809]	0.649 [0.638, 0.659]	0.615 [0.600, 0.628]	0.856 [0.851, 0.863]
Initial	0.923 [0.919, 0.928]	0.000 [0.000, 0.000]	0.000 [0.000, 0.000]	0.000 [0.000, 0.000]	0.500 [0.500, 0.500]	0.077 [0.072, 0.081]	0.000 [0.000, 0.000]	0.923 [0.919, 0.928]
Recurrent	0.732 [0.723, 0.741]	0.770 [0.760, 0.781]	0.830 [0.821, 0.842]	0.799 [0.790, 0.807]	0.694 [0.687, 0.703]	0.748 [0.738, 0.758]	0.403 [0.387, 0.420]	0.647 [0.632, 0.669]
Baseline								
All	0.858 [0.852, 0.863]	0.776 [0.765, 0.787]	0.823 [0.813, 0.834]	0.799 [0.790, 0.807]	0.932 [0.928, 0.936]	0.887 [0.879, 0.893]	0.689 [0.677, 0.700]	0.905 [0.899, 0.910]
Initial	0.914 [0.908, 0.920]	0.505 [0.464, 0.549]	0.382 [0.345, 0.418]	0.435 [0.399, 0.469]	0.873 [0.860, 0.885]	0.466 [0.429, 0.501]	0.393 [0.358, 0.428]	0.943 [0.937, 0.948]
Recurrent	0.793 [0.783, 0.802]	0.805 [0.793, 0.815]	0.895 [0.886, 0.904]	0.847 [0.839, 0.855]	0.872 [0.864, 0.879]	0.924 [0.918, 0.929]	0.537 [0.515, 0.558]	0.765 [0.746, 0.783]
Single-task								
All	NA	NA	NA	NA	NA	NA	NA	NA
Initial	0.889 [0.882, 0.896]	0.400 [0.372, 0.432]	**0.552 [0.518, 0.589]**	0.464 [0.436, 0.493]	0.870 [0.857, 0.882]	0.455 [0.416, 0.493]	0.411 [0.380, 0.441]	**0.956 [0.951, 0.960]**
Recurrent	0.794 [0.784, 0.804]	0.795 [0.783, 0.807]	0.922 [0.913, 0.929]	0.854 [0.846, 0.862]	0.873 [0.865, 0.882]	0.928 [0.921, 0.934]	0.529 [0.507, 0.551]	0.791 [0.771, 0.810]
With IDH occurrence info. (binary)								
All	0.870 [0.865, 0.876]	**0.799 [0.788, 0.810]**	0.831 [0.821, 0.841]	0.815 [0.807, 0.823]	0.941 [0.938, 0.944]	0.900 [0.894, 0.906]	0.715 [0.704, 0.727]	0.910 [0.904, 0.915]
Initial	0.925 [0.919, 0.930]	0.589 [0.545, 0.631]	0.438 [0.402, 0.472]	0.502 [0.467, 0.534]	0.900 [0.888, 0.911]	0.541 [0.503, 0.579]	0.469 [0.431, 0.503]	0.948 [0.942, 0.953]
Recurrent	0.809 [0.798, 0.818]	0.822 [0.811, 0.833]	0.895 [0.886, 0.904]	0.857 [0.849, 0.865]	0.885 [0.877, 0.893]	0.932 [0.926, 0.938]	0.574 [0.553, 0.594]	0.777 [0.759, 0.794]
With detailed IDH occurrence information								
All	**0.878 [0.873, 0.883]**	0.792 [0.781, 0.802]	**0.875 [0.866, 0.884]**	**0.831 [0.824, 0.839]**	**0.951 [0.948, 0.954]**	**0.917 [0.911, 0.923]**	**0.739 [0.728, 0.749]**	**0.931 [0.926, 0.936]**
Initial	**0.926 [0.921, 0.932]**	0.587 [0.549, 0.626]	0.513 [0.477, 0.550]	**0.548 [0.517, 0.580]**	**0.913 [0.903, 0.923]**	**0.565 [0.530, 0.603]**	**0.509 [0.477, 0.543]**	0.954 [0.950, 0.959]
Recurrent	0.823 [0.815, 0.832]	0.817 [0.807, 0.829]	**0.933 [0.926, 0.940]**	0.871 [0.865, 0.879]	0.909 [0.903, 0.916]	0.948 [0.943, 0.952]	0.607 [0.589, 0.626]	**0.839 [0.823, 0.856]**
Loss weighting								
All	NA	NA	NA	NA	NA	NA	NA	NA
Initial	0.919 [0.913, 0.924]	0.537 [0.496, 0.576]	0.469 [0.433, 0.505]	0.500 [0.468, 0.533]	0.887 [0.875, 0.899]	0.510 [0.471, 0.547]	0.458 [0.422, 0.492]	0.950 [0.945, 0.954]
Recurrent	0.807 [0.798, 0.816]	0.810 [0.799, 0.820]	0.914 [0.906, 0.922]	0.859 [0.851, 0.866]	0.892 [0.885, 0.899]	0.938 [0.932, 0.943]	0.569 [0.547, 0.588]	0.801 [0.782, 0.818]
Loss weighting with detailed IDH occurrence info.								
All	NA	NA	NA	NA	NA	NA	NA	NA
Initial	0.925 [0.920, 0.931]	**0.635 [0.588, 0.685]**	0.323 [0.290, 0.360]	0.428 [0.393, 0.467]	0.910 [0.899, 0.920]	0.530 [0.491, 0.569]	0.418 [0.382, 0.457]	0.939 [0.933, 0.944]
Recurrent	**0.841 [0.832, 0.850]**	**0.843 [0.833, 0.853]**	0.924 [0.917, 0.931]	**0.882 [0.874, 0.888]**	**0.920 [0.914, 0.927]**	**0.954 [0.950, 0.959]**	**0.647 [0.629, 0.665]**	0.836 [0.820, 0.851]

Fall20, a decrease in SBP ≥20 mm Hg from baseline; IDH: Intradialytic Hypotension; AUROC: Area Under the Receiver Operating Characteristic curve; AUPRC: Area Under the Precision-Recall Curve; MCC: Matthews Correlation Coefficient; NPV: Negative Predictive Value; NA: Not Applicable; info.: information.

† Bold values indicate the highest performance, while underlined values show the second-best performance for each metric.

Supplementary Table S7. Predictive performance of baseline model on the definition of Fall30

Model types	Performance metrics							
IDH occurrence type	Accuracy	Precision	Recall	F1	AUROC	AUPRC	MCC	NPV
Naïve baseline								
All	0.881 [0.876, 0.885]	0.722 [0.708, 0.732]	0.645 [0.631, 0.656]	0.681 [0.669, 0.690]	0.792 [0.785, 0.798]	0.536 [0.520, 0.549]	0.610 [0.596, 0.620]	0.914 [0.910, 0.918]
Initial	0.952 [0.949, 0.956]	0.000 [0.000, 0.000]	0.000 [0.000, 0.000]	0.000 [0.000, 0.000]	0.500 [0.500, 0.500]	0.048 [0.044, 0.051]	0.000 [0.000, 0.000]	0.952 [0.949, 0.956]
Recurrent	0.715 [0.702, 0.725]	0.722 [0.708, 0.732]	0.809 [0.794, 0.821]	0.763 [0.751, 0.771]	0.700 [0.685, 0.712]	0.693 [0.678, 0.704]	0.412 [0.383, 0.436]	0.701 [0.683, 0.724]
Baseline								
All	0.896 [0.891, 0.901]	0.725 [0.710, 0.741]	0.765 [0.750, 0.780]	0.745 [0.733, 0.757]	0.940 [0.936, 0.945]	0.824 [0.811, 0.836]	0.680 [0.666, 0.695]	0.941 [0.937, 0.945]
Initial	0.947 [0.943, 0.952]	0.510 [0.461, 0.557]	0.361 [0.322, 0.398]	0.422 [0.383, 0.460]	0.896 [0.883, 0.909]	0.438 [0.397, 0.481]	0.402 [0.362, 0.439]	0.965 [0.961, 0.968]
Recurrent	0.769 [0.756, 0.782]	0.758 [0.741, 0.773]	0.872 [0.859, 0.886]	0.811 [0.799, 0.823]	0.856 [0.844, 0.866]	0.887 [0.875, 0.898]	0.526 [0.501, 0.551]	0.790 [0.769, 0.811]
Single-task								
All	NA	NA	NA	NA	NA	NA	NA	NA
Initial	0.934 [0.929, 0.939]	0.411 [0.376, 0.448]	**0.525 [0.488, 0.566]**	0.461 [0.428, 0.496]	0.905 [0.893, 0.917]	0.433 [0.391, 0.477]	0.430 [0.395, 0.466]	**0.973 [0.970, 0.976]**
Recurrent	0.795 [0.782, 0.808]	0.778 [0.763, 0.794]	0.902 [0.890, 0.913]	0.836 [0.825, 0.847]	0.876 [0.866, 0.887]	0.905 [0.894, 0.916]	0.577 [0.550, 0.604]	0.828 [0.808, 0.849]
With IDH occurrence info. (binary)								
All	0.905 [0.900, 0.910]	0.752 [0.736, 0.767]	0.778 [0.763, 0.793]	0.765 [0.753, 0.777]	0.950 [0.946, 0.954]	0.844 [0.833, 0.856]	0.706 [0.691, 0.720]	0.945 [0.941, 0.949]
Initial	0.953 [0.949, 0.957]	0.598 [0.545, 0.642]	0.379 [0.341, 0.416]	0.464 [0.425, 0.501]	0.920 [0.909, 0.930]	0.487 [0.443, 0.528]	0.453 [0.414, 0.491]	0.966 [0.962, 0.969]
Recurrent	0.788 [0.776, 0.800]	0.775 [0.759, 0.791]	0.882 [0.870, 0.895]	0.825 [0.814, 0.836]	0.872 [0.862, 0.882]	0.901 [0.890, 0.910]	0.566 [0.542, 0.591]	0.811 [0.792, 0.832]
With detailed IDH occurrence information								
All	0.911 [0.907, 0.916]	0.761 [0.746, 0.776]	**0.804 [0.790, 0.819]**	**0.782 [0.770, 0.794]**	**0.958 [0.955, 0.961]**	**0.864 [0.854, 0.874]**	**0.727 [0.713, 0.741]**	**0.951 [0.947, 0.955]**
Initial	0.950 [0.946, 0.954]	0.538 [0.496, 0.585]	0.459 [0.419, 0.498]	**0.495 [0.458, 0.533]**	0.930 [0.920, 0.939]	0.489 [0.449, 0.536]	**0.471 [0.433, 0.510]**	0.970 [0.966, 0.973]
Recurrent	0.817 [0.805, 0.828]	0.808 [0.792, 0.822]	0.891 [0.878, 0.903]	0.847 [0.837, 0.857]	0.901 [0.892, 0.909]	0.920 [0.911, 0.928]	0.626 [0.601, 0.649]	0.834 [0.816, 0.851]
Loss weighting								
All	NA	NA	NA	NA	NA	NA	NA	NA
Initial	0.943 [0.938, 0.947]	0.459 [0.416, 0.502]	0.403 [0.362, 0.443]	0.429 [0.392, 0.466]	0.890 [0.877, 0.902]	0.400 [0.359, 0.444]	0.400 [0.362, 0.438]	0.967 [0.963, 0.970]
Recurrent	0.790 [0.778, 0.803]	0.792 [0.776, 0.808]	0.855 [0.841, 0.869]	0.822 [0.811, 0.835]	0.875 [0.865, 0.886]	0.902 [0.892, 0.913]	0.569 [0.544, 0.595]	0.787 [0.768, 0.806]
Loss weighting with detailed IDH occurrence info.								
All	NA	NA	NA	NA	NA	NA	NA	NA
Initial	0.952 [0.949, 0.956]	0.574 [0.531, 0.623]	0.417 [0.377, 0.455]	0.483 [0.444, 0.521]	**0.933 [0.924, 0.941]**	**0.491 [0.452, 0.531]**	0.465 [0.425, 0.504]	0.968 [0.964, 0.971]
Recurrent	**0.828 [0.817, 0.839]**	**0.812 [0.798, 0.826]**	**0.909 [0.897, 0.919]**	**0.858 [0.848, 0.867]**	**0.919 [0.912, 0.927]**	**0.937 [0.930, 0.945]**	**0.650 [0.628, 0.671]**	**0.857 [0.840, 0.872]**

Fall30, a decrease in SBP ≥30 mm Hg from baseline; IDH: Intradialytic Hypotension; AUROC: Area Under the Receiver Operating Characteristic curve; AUPRC: Area Under the Precision-Recall Curve; MCC: Matthews Correlation Coefficient; NPV: Negative Predictive Value; NA: Not Applicable; info.: information.

† Bold values indicate the highest performance, while underlined values show the second-best performance for each metric.

Supplementary Table S7. Average number of recurrent intradialytic hypotension (IDH) events per session stratified by baseline systolic blood pressure (SBP)

IDH definitions	Average number of IDH recurrences among sessions with IDH occurrence	
	Higher baseline SBP	Lower baseline SBP
Nadir90	0.073 ± 0.387	0.285 ± 1.036
Nadir100	0.174 ± 0.716	0.795 ± 2.006
Fall20/MAP10	5.291 ± 3.632	1.860 ± 2.733
Fall20	4.797 ± 3.659	1.351 ± 2.354
Fall30	3.091 ± 3.428	0.533 ± 1.472

Nadir90, nadir systolic blood pressure (SBP) <90 mm Hg; Nadir100, nadir SBP <100 mm Hg; Fall20/MAP10, a decrease in SBP by ≥20 mm Hg or a decrease in mean arterial pressure >10 mm Hg from baseline; Fall20, a decrease in SBP ≥20 mm Hg from baseline; Fall30, a decrease in SBP ≥30 mm Hg from baseline.

REFERENCES

1. Zhang, H., et al., *Real-time prediction of intradialytic hypotension using machine learning and cloud computing infrastructure.* Nephrology Dialysis Transplantation, 2023. **38**(7): p. 1761-1769.
2. Rocha, A., et al., *Effect of dialysis day on intradialytic hypotension risk.* Kidney and Blood Pressure Research, 2016. **41**(2): p. 168-174.
3. Liu, K.-H., et al., *Ambient temperature and the occurrence of intradialytic hypotension in patients receiving hemodialysis.* Clinical Kidney Journal, 2024. **17**(1): p. sfad304.
